# STIM2 regulates both intracellular Ca^2+^ distribution and Ca^2+^ movement in skeletal myotubes

**DOI:** 10.1038/s41598-017-18256-3

**Published:** 2017-12-20

**Authors:** Mi Ri Oh, Keon Jin Lee, Mei Huang, Jin Ock Kim, Do Han Kim, Chung-Hyun Cho, Eun Hui Lee

**Affiliations:** 1 Department of Physiology, College of Medicine, The Catholic University of Korea, Seoul, 06591 Republic of Korea; 2 School of Life Sciences, GIST, Gwangju, 61005 Republic of Korea; 3 Department of Pharmacology, College of Medicine, Seoul National University, Seoul, 08826 Republic of Korea

## Abstract

Stromal interaction molecule 1 (STIM1) along with Orai1 mediates extracellular Ca^2+^ entry into the cytosol through a store-operated Ca^2+^ entry (SOCE) mechanism in various tissues including skeletal muscle. However, the role(s) of STIM2, a homolog of STIM1, in skeletal muscle has not been well addressed. The present study, first, was focused on searching for STIM2-binding proteins from among proteins mediating skeletal muscle functions. This study used a binding assay, quadrupole time-of-flight mass spectrometry, and co-immunoprecipitation assay with *bona-fide* STIM2- and SERCA1a-expressing rabbit skeletal muscle. The region for amino acids from 453 to 729 of STIM2 binds to sarcoplasmic/endoplasmic reticulum Ca^2+^-ATPase 1a (SERCA1a). Next, oxalate-supported ^45^Ca^2+^-uptake experiments and various single-myotube Ca^2+^ imaging experiments using STIM2-knockdown mouse primary skeletal myotubes have suggested that STIM2 attenuates SERCA1a activity during skeletal muscle contraction, which contributes to the intracellular Ca^2+^ distribution between the cytosol and the SR at rest. In addition, STIM2 regulates Ca^2+^ movement through RyR1 during skeletal muscle contraction as well as SOCE. Therefore, via regulation of SERCA1a activity, STIM2 regulates both intracellular Ca^2+^ distribution and Ca^2+^ movement in skeletal muscle, which makes it both similar to, yet different from, STIM1.

## Introduction

Contraction and relaxation are the main tasks of skeletal muscle^1–5^. Skeletal muscle contraction occurs via excitation-contraction (EC) coupling. During EC coupling, the action potential in skeletal muscle cells (also called muscle fiber or myotubes) by neural stimulation spreads along the surface of the plasma membrane and to the interior of skeletal muscle cells via transverse (t)-tubules, which activate dihydropyridine receptors (DHPRs, membrane voltage-sensors) in the t-tubule membrane. Subsequently, RyR1 (an internal Ca^2+^-releasing channel) in the sarcoplasmic reticulum (SR) membrane is activated by the active DHPRs via physical interactions, which allows Ca^2+^ release from the SR to the cytosol via the RyR1 for skeletal muscle contraction (up to sub-micromolar concentrations of Ca^2+^ in the cytosol). Ca^2+^-uptake from the cytosol to the SR is a critical step for skeletal muscle relaxation^3,4,6^. Sarcoplasmic/endoplasmic reticulum Ca^2+^-ATPase 1a (SERCA1a, the major isoform in adult skeletal muscle) is a Ca^2+^ pump in the SR membrane and uptakes Ca^2+^ from the cytosol into the SR to reduce cytosolic Ca^2+^ levels to that at rest (nanomolar range) and to refill the SR with Ca^2+^ during skeletal muscle relaxation^7–9^. Therefore, Ca^2+^ in the SR is the major source of Ca^2+^ for skeletal muscle contraction, and spatial and temporal distribution of the intracellular Ca^2+^ between the SR and the cytosol via RyR1, and SERCA1 is a key factor for the cycling of skeletal muscle contraction and relaxation^1–6^. Patients with Brody syndrome suffer from exercise-induced muscle stiffness and delayed muscle relaxation due to a reduction in SERCA1a activity, however, the reduced SERCA1a activity does not involve a mutation in the SERCA1a gene^10–12^. This suggests that SERCA1 is not the only cause of Brody syndrome, and regulatory proteins of SERCA1a activity could be candidates for the diagnosis or treatment of patients with Brody syndrome. The DHPR, RyR1, and SERCA1a have a functionally efficient arrangement in the t-tubule and the SR membranes that are closely juxtaposed (known as the triad junction or junctional membrane complex)^13–15^. Junctophilin (JP) is required for the proper formation of the junctional membrane complex^15,16^.

Store-operated Ca^2+^ entry (SOCE) is a ubiquitous Ca^2+^ entry way in various cells such as skeletal muscle cells, and stromal interaction molecule 1 (STIM1, a Ca^2+^ sensor in ER/SR membranes), and Orai1 (a Ca^2+^ entry channel in t-tubule/plasma membranes) are the main proteins responsible for SOCE^17,18^. SOCE is required for replenishing the ER/SR with Ca^2+^ and sustaining the presence of a certain concentration of Ca^2+^ in the cytosol. In general, during SOCE, the depletion of Ca^2+^ in the ER induces the dissociation of Ca^2+^ from STIM1, which allows the relocation of STIM1 to the ER membranes near plasma membranes, the interaction of STIM1 with Orai1 (called puncta), and extracellular Ca^2+^ entry through Orai1^19–23^. The formation of puncta in skeletal muscle occurs as a part of the terminal differentiation of myoblasts (proliferative and undifferentiated skeletal muscle cells) to form mature myotubes in an SR depletion-independent manner, which results in SOCE that is more rapid in skeletal muscle than in other cells, as it occurs in a matter of seconds^15,24–26^.

STIM1 has a short intraluminal N-terminus that contains an actual EF-hand domain and a sterile α-motif (SAM) domain, a single-transmembrane domain, and a cytosolic C-terminus that contains three coiled-coil domains, SOAR (STIM1-Orai1-activating region)/CAD (Ca^2+^ release-activated Ca^2+^ (CRAC)-activating domain) domain, an ezrin-radixin-moesin domain, a proline/serine-rich domain, and a lysine-rich domain^27–30^. The EF-hand domain is a Ca^2+^-sensing region^26,31,32^. The EF-SAM domain is responsible for the self-oligomerization and relocalization of STIM1s to form puncta^20,21^. The first coiled-coil domain participates in the self-oligomerization of STIM1 at rest^33^. The CAD/SOAR domain physically interacts with, and activates, Orai1^34,35^. The CAD also participates in the self-oligomerization of STIM1^33^. The lys-rich domain is responsible for the Orai1-independent plasma membrane targeting of STIM1^35^. STIM2 is a homolog of STIM1 and is widely expressed in many types of tissues, although the expression level of STIM2 is usually lower than that of STIM1^29^. The role of STIM2 in SOCE is less clear because initial studies on the role of STIM2 in SOCE using smooth muscle cells or heterologous expression systems have produced contradictory results: a positive role in SOCE^36^, or no effect on SOCE^37,38^. STIM2 has an EF-hand region that is more sensitive to Ca^2+^ than STIM1^39,40^.

The conditional deletion of STIM1 in skeletal muscle is prenatally lethal in mice due to myopathy and defective muscle differentiation^41,42^. Knockdown of STIM1 in human skeletal muscle reduces SOCE, the degree of the terminal differentiation to myotubes, the cytosolic Ca^2+^ level at rest, and the amount of Ca^2+^ in the SR^43,44^. Previously, we reported that STIM1 is involved in intracellular Ca^2+^ release during skeletal EC coupling by negatively regulating RyR1 activity via a direct interaction with DHPR^26^. We also found that the C-terminal region of STIM1 between amino acids from 449 to 671 binds to SERCA1a, and the binding is required for the full activity of SERCA1a during skeletal muscle relaxation^45^. Involvement of STIM1 in the human skeletal muscle diseases has been reported. Patients with a deficiency in STIM1 show muscular hypotonia^46^. Patients with one of four STIM1 missense mutations in EF-hand (constitutively active STIM1 mutants, H72Q, D84G, H109N or H109R) show muscular atrophy, tubular aggregate myopathy, and/or progressive muscle weakness^47^. Patients with a loss-of-function mutation of STIM1, E136X, show congenital myopathies as well as severe combined immunodeficiency^46,48,49^. Patients with another STIM1 mutation, R429C, also show muscular hypotonia^50^. Muscle fibers from *mdx* mice, an animal model for Duchenne muscular dystrophy that is characterized by progressive muscle weakness, show increases in both STIM1 expression and SOCE^51,52^. It is possible that STIM2 could be also related to the skeletal muscle diseases because STIM2 shares high homologies with STIM1 in their amino acid sequences and domains although there has yet been no report on the direct relevance of STIM2 to skeletal muscle diseases. In case of the smooth muscle, STIM2 contributes to the proliferation of pulmonary arterial smooth muscle cells in patients with pulmonary arterial hypertension^37,53,54^.

Considering that STIM1 plays important and various roles in skeletal muscle functions and diseases^26,41–46,48,50–52^, it is possible that STIM2 could also participate in unique functions of skeletal muscle such as terminal differentiation, Ca^2+^ movements during contraction and relaxation, and more. However, until now, the role of STIM2 in skeletal muscle has not been well addressed. Therefore, in the present study, biochemical approaches were used to identify STIM2-binding proteins from among the proteins that mediate skeletal muscle function. We found that STIM2 binds to SERCA1a, and the functional contribution of the binding between STIM2 and SERCA1a in skeletal muscle was examined using mouse primary skeletal myotubes (instead of a heterologous expression system involving variations in expression) and single-myotube Ca^2+^-imaging experiments.

## Results

### In skeletal muscle, STIM2 binds to SERCA1a via a region of amino acids from 453 to 729

**Figure 1:**
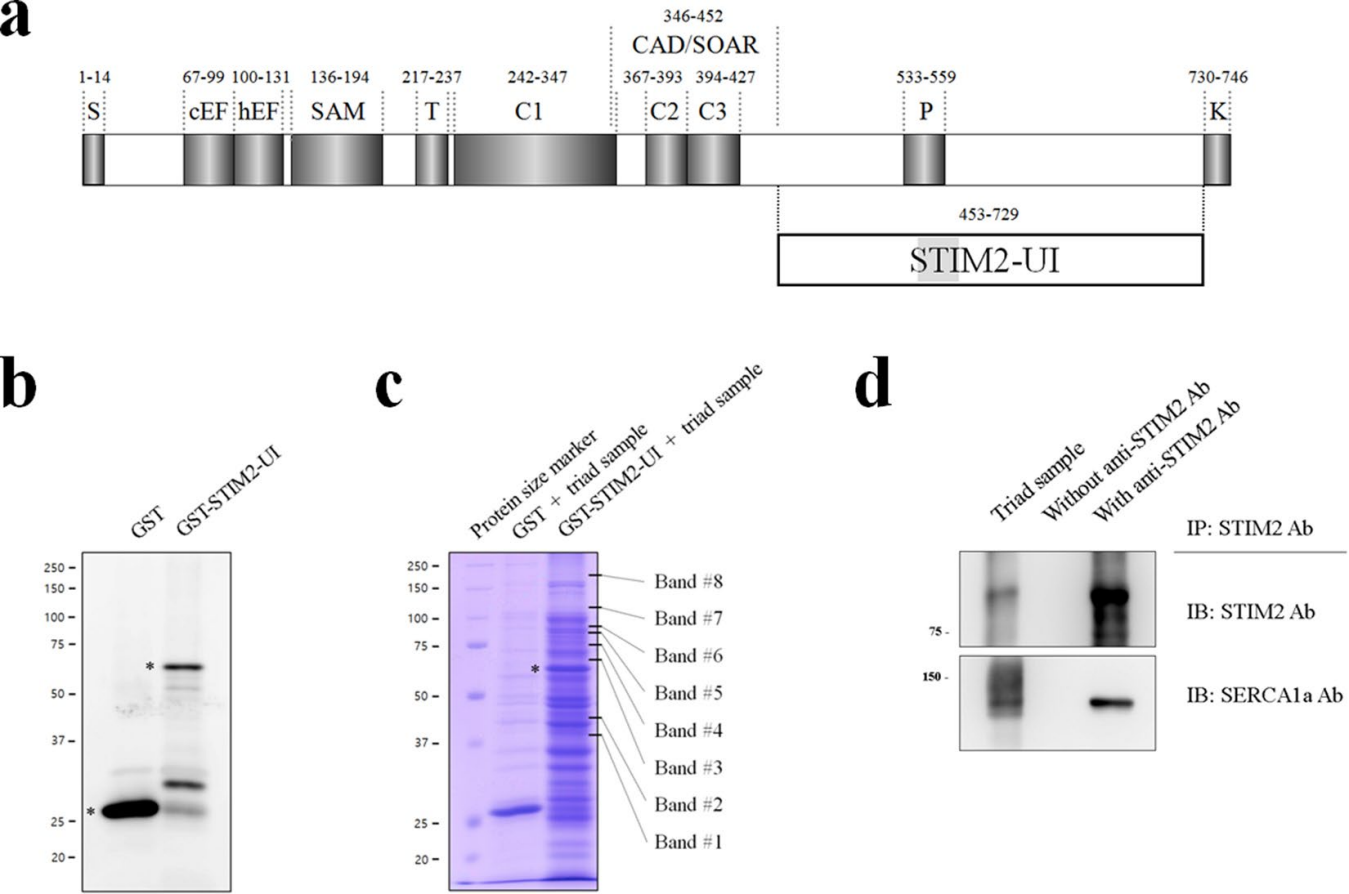
Schematic primary sequences of the STIM2 and STIM2-UI region, binding assay of GST-fused STIM1-UI protein with a triad sample, and co-immunoprecipitation of full-length STIM2 with SERCA1a. (**a**) The position of each domain in STIM2 is presented according to previous reports: the overall diagram^92,93^, and CAD/SOAR^94^. Numbers indicate the sequence of amino acids. UI means unidentified region. S, signal peptide; cEF, canonical EF-hand; hEF, non-functional hidden EF-hand; SAM, sterile α-motif; T, transmembrane domain; C, coiled-coil domain; CAD/SOAR, Ca^2+^ release-activated Ca^2+^ (CRAC)-activating domain/STIM1-Orai1-activating region; P, proline/serine-rich domain; K, lysine-rich domain. (**b**) Immobilized GST-fused STIM2-UI proteins on GST beads were separated on a 10% SDS-PAGE gel, and the gel was subjected to immunoblot with anti-GST antibody. GST or GST-fused STIM2-UI proteins are indicated by asterisks. (**c**) The bound proteins in the binding assay of GST-fused STIM2-UI protein with the triad sample from rabbit skeletal muscle were separated on a 10% SDS-PAGE gel, and the gel was stained with Coomassie Blue. GST was used as a negative control. GST-fused STIM2-UI proteins are indicated by an asterisk. The eight proteins that were bound to the GST-fused STIM2-UI protein are indicated on the right side of the figure (bands 1 to 8). (**d**) The triad sample obtained from rabbit skeletal muscle (30 μg of total proteins) was subjected to a co-immunoprecipitation assay with anti-STIM2 antibody, and the immunoprecipitant was subjected to immunoblot analysis with anti-STIM2 or anti-SERCA1a antibodies. Triad sample indicates a simple immunoblot of the triad sample. Without Ab indicates a reaction without anti-STIM2 antibody. Three independent experiments were conducted. IB, IP, or Ab means immunoblot, immunoprecipitation, or antibody, respectively. The immunoblot data were cropped from the immunoblot images of different gels and were grouped. The full-length blots are presented in Supplementary Fig. 4. SERCA1a was successfully co-immunoprecipitated with full-length STIM2.

To find unique STIM2-binding proteins among proteins mediating or regulating the contraction and relaxation of skeletal muscle, first, cDNA of the variable region between STIM1 and STIM2 (amino acids from 453 to 729 in STIM2, i.e., STIM2-UI in Fig. 1a) was constructed in a GST vector. The region was referred to as STIM2-UI because the role of the region was unidentified (UI). The GST-fused STIM2-UI protein was expressed in E. coli. The bacterial cell lysate was subjected to immunoblot assay with anti-GST antibody (Fig. 1b). The GST-fused STIM2-UI protein was successfully expressed (approximately 56 kDa). Next, affinity beads were prepared by immobilizing the GST-fused STIM2-UI proteins on GST beads, and the affinity beads were incubated with the solubilized triad sample from ‘rabbit’ skeletal muscle (i.e., binding assay). The triad sample is an enriched one with triad proteins that mediate the contraction and relaxation of skeletal muscle, as described in the Materials and Methods section. The proteins that were bound to the affinity beads were separated on a SDS-PAGE gel and were stained with Coomassie Brilliant Blue in order to find the proteins that could specifically bind to the GST-fused STIM2-UI protein (Fig. 1c). The bands for the proteins that bound to GST itself were excluded from consideration. Eight bands appeared as proteins that were bound to the GST-fused STIM2-UI protein.

In identifying the binding proteins, the eight bands were subjected to in-gel digestion and to qTOF mass spectrometry (qTOP-MS). Supplementary Fig. 1 and Table 1 show the results of qTOF-MS and database searches. Bands 1 to 6 were identified as non-specifically bound proteins that originated from E. coli. Band 7 was identified as SERCA1a that originated from rabbit skeletal muscle, which suggested that SERCA1a could be a STIM2-binding protein in skeletal muscle. The binding of full-length STIM2 to SERCA1a was accessed using *bona-fide* STIM2- and SERCA1a-expressing rabbit skeletal muscle and co-immunoprecipitation assay, and SERCA1a was successfully co-immunoprecipitated with full-length STIM2 (Fig. 1d). Therefore, based on the two different approaches (binding assay and qTOF-MS, and co-immunoprecipitation assays), we suggest that STIM2 binds to SERCA1a in skeletal muscle via its region from amino acids from 453 to 729.

**Table 1:** List of proteins identified by qTOF-MS.

Band #	Protein name	Mascot ID	Monoisotopic mass	Species	Matching score	Matching peptides
1	Fructose-bisphosphate aldolase class 2	ALF_ECOLI	39355	E. coli	112	AFQELNAIDVL (617.32) IFDFVKPGVITGDDVQK (626.67) VKAPVIVQFSNGGASFIAGK (664.37) SKIFDFVKPGVITGDDVQK (698.37) FTIAASFGNVHGVYKPGNVVLTPTILR (718.65)
2	3-oxoacyl-[acyl-carrier-protein] synthase 1	FABB_ECOLI	42934	E. coli	124	AVITGLGIVSSIGNNQQEVLASLR (813.78)
3	Peptidyl-prolyl cis-trans isomerase D	PPID_ECOLI	68108	E. coli	90	ALDAYYALQQK (642.33) LIDEALLDQYAR (710.37) QAIFATPAFQVDGK (746.89)
4	GTP-binding protein TypA/BipA	TYPA_ECOLI	67546	E. coli	200	EGFELAVSRPK (616.83) INIVDTPGHADFGGEVER (642.65) AVAFALFGLQDR (654.35) ASGTDEAVVLVPPIR (762.42)
5	Chaperone protein ClpB	CLPB_ECOLI	95700	E. coli	180	VIGQNEAVDAVSNAIRR (604.65) ELVLGVVSHNFRPEFINR (709.38) LVGAPPGYVGYEEGGYLTEAVR (766.39) VIGQNEAVDAVSNAIR (828.44) VFVAEPSVEDTIAILR (879.98)
6	Phenylalanine-tRNA ligase beta subunit	SYFB_ECOLI	88077	E. coli	212	FVPDTQAPLGIR (657.36) SLAISLILQDTSR (708.90) IGFVGVVHPELER (726.40) VAVATIGAVLPGDFK (729.41) VYGYNNIPDEPVQASLIMGTHR (830.74)
7	Sarcoplasmic/endoplasmic reticulum Ca^2+^-ATPase 1a (SERCA1a)	AT2A1_MOUSE	110747	Mouse	121	VGEATETALTTLVEK (781.42)
8	No matching signal					

It is possible that, according to the expected protein size, band 8 could be responsible for an oligomeric STIM2-UI (approximately 224 kDa in the case of a tetramer). However, there was no matching signal to the band 8 in the known databases, suggesting that the STIM2-UI region is irrelevant to the self-oligomerization of STIM2. It is possible that the signal from the band 8 could be obtained from experimental shortcomings, for example, an incomplete digestion of the band 8 by trypsin, which could result in a mixed signal that does not match to the known databases.

### STIM2 in skeletal myotubes attenuates SERCA1a activity, which contributes to the Ca^2+^ distribution between the cytosol and the SR

**Figure 2:**
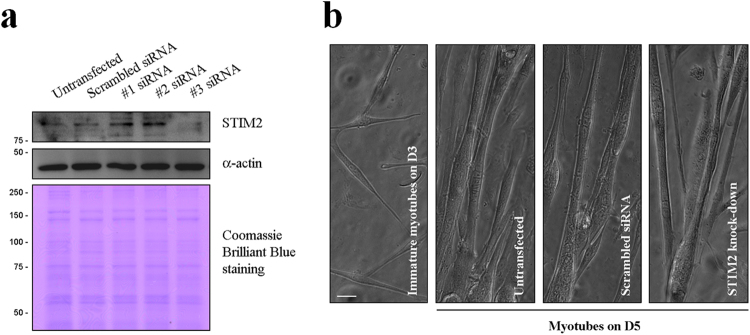
Knockdown of STIM2 in mouse primary skeletal myotubes. (**a**) Three different forms of siRNA were used to knock down STIM2 in mouse primary skeletal myotubes. The lysate of the STIM2-knockdown myotubes (50 μg of total protein) was subjected to immunoblot analysis with anti-STIM2 antibody (upper panel). α-actin and Coomassie Brilliant Blue staining were loading controls. #3 siRNA knocked down STIM2 more effectively compared with others (up to 90%). Untransfected control and scrambled siRNA-transfected myotubes were used as negative controls. The immunoblot data were cropped from the immunoblot images of different gels and were grouped. The full-length blots are presented in Supplementary Fig. 5. Three independent experiments were conducted. (**b**) The STIM2-knockdown myotubes with #3 siRNA show normal myotube formations, and are indistinguishable from the untransfected or the scrambled siRNA-transfected control. ‘Immature myotubes on D3’ means untransfected immature myotubes on differentiation day 3 (left). ‘Myotubes on D5’ means untransfected, scrambled siRNA-transfected, or STIM2-knockdown myotubes on differentiation day 5. The bar represents 50 μm.

To examine how STIM2 is related to SERCA1a activity, STIM2 in mouse primary skeletal myotubes was knocked down using siRNA. Three different siRNAs for mouse STIM2 were used to knock down STIM2 (Table 2, upper panel). Immunoblot assay with anti-STIM2 antibody using the lysate of STIM2-knockdown myotubes by each siRNA showed that STIM2 expression was reduced up to 90% by #3 siRNA compared with that of the untransfected or scrambled siRNA control (Fig. 2a). Therefore, #3 siRNA was used for the further experiments to knock down STIM2. On the other hand, fully differentiated myotubes on differentiation day 5 were much longer and thicker and more multi-nucleated than the untransfected immature myotubes on differentiation day 3 (left image of Fig. 2b). siRNA-transfected myotubes were not distinguishable from the untransfected or scrambled siRNA-transfected control. This suggests that myotube formation (i.e., the terminal differentiation) was not significantly affected by the STIM2-knockdown, and STIM2 is not significantly involved in the terminal differentiation of skeletal muscle.

**Table 2:** Sequences of siRNAs used for knocking down STIM2, and PCR primers for the construction of GST-fused STIM2-UI region.

**siRNA**	**Sense**	**Antisense**
Scrambled siRNA	5′-ACGUGACACGUUCGGAGAAUU-3′	5′-UUCUCCGAACGUGUCACGUUU-3′
#1 siRNA	5′-UGCCACAAUAUGAGAAGAAUU-3′	5′-UUCUUCUCAUAUUGUGGCAUU-3′
#2 siRNA	5′-AUCGGAACGAAGAGGAGGAUU-3′	5′-UCCUCCUCUUCGUUCCGAUUU-3′
#3 siRNA	5′-AAUGUUUCCAGAGUAAGCAUU-3′	5′-UGCUUACUCUGGAAACAUUUU-3′
**PCR primers for the construction of GST-fused STIM2-UI region**		
Forward primer (with EcoR1 enzyme site)		5′-CGGAATTCATGAGCCTGACCTCTTCCCTTTATTC-3′
Backward primer (with Sal1 enzyme site)		5′-CGTCGACTCACTCTCCATTATGACAAAGGTCATG-3′

**Figure 3:**
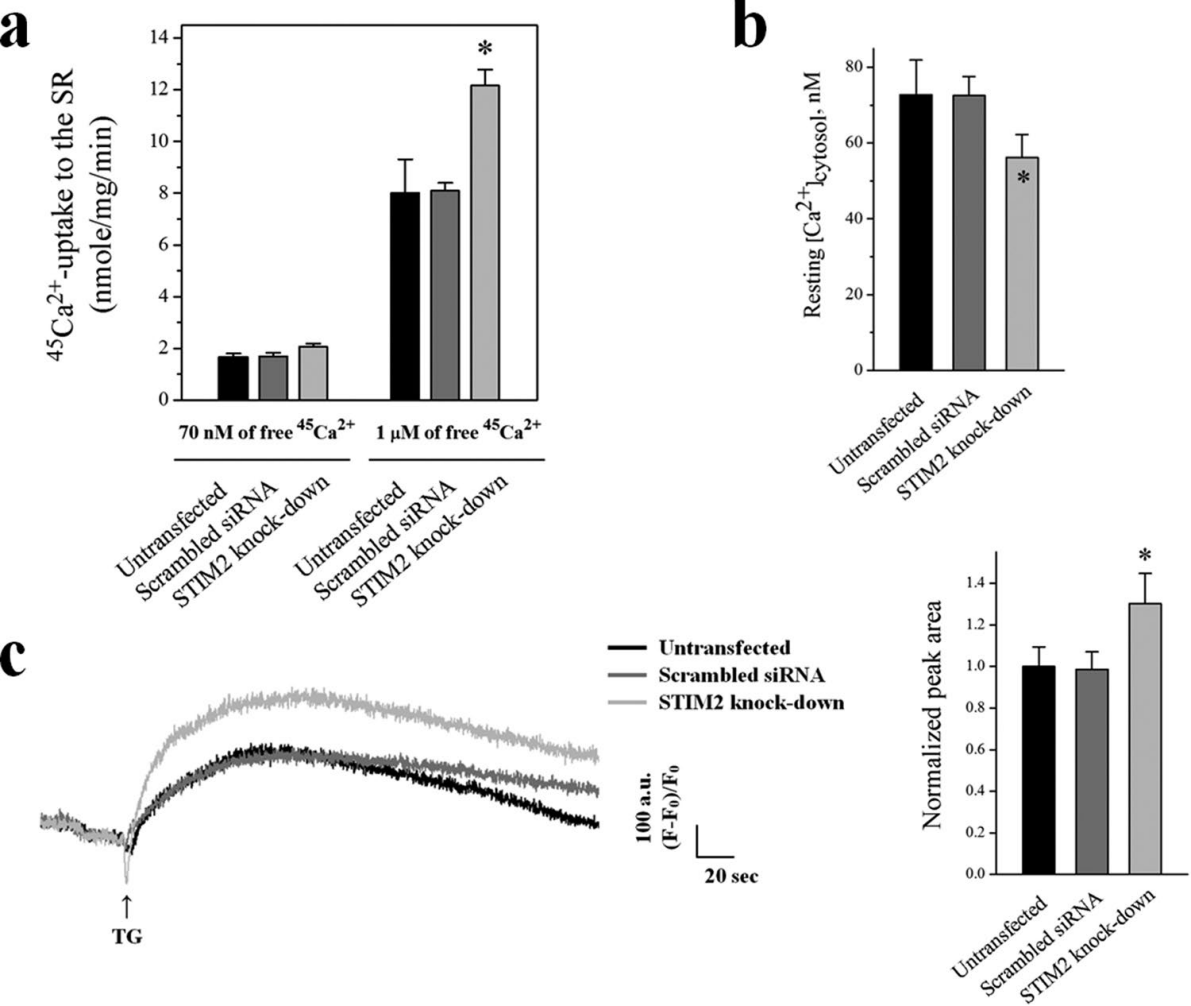
Increases in ^45^Ca^2+^-uptake into the SR through SERCA1a and the amount of Ca^2+^ in the SR, and a decrease in the cytosolic Ca^2+^ level at rest by STIM2-knockdown. (**a**) Oxalate-supported ^45^Ca^2+^-uptake into the SR through SERCA1a using the homogenate of the STIM2-knockdown myotubes was measured at 70 nM or 1 μM of free ^45^Ca^2+^. Either an untransfected or a scrambled siRNA-transfected control was used as a negative control. The results are presented as the mean ± S.E. of three independent experiments (Table 3). *Significant difference compared with the untransfected control (*p* < 0.05). The STIM2-knockdown myotubes showed a significantly increased ^45^Ca^2+^-uptake through SERCA1a only at 1 μM of free ^45^Ca^2+^. (**b**) Cytosolic Ca^2+^ level at rest was examined in the STIM2-knockdown myotubes, and histograms are shown for the mean values of each. *Significant difference compared with the untransfected control (*p* < 0.05). The values are presented as the mean ± S.E. for the number of myotubes shown in the parentheses of Table 4. Cytosolic Ca^2+^ level at rest was decreased by STIM2-knockdown. (**c**) To measure the amount of Ca^2+^ in the SR, TG was applied to the STIM2-knockdown myotubes in the absence of extracellular Ca^2+^ to avoid extracellular Ca^2+^ entry. A representative trace for each group is shown, and the results are summarized as bar graphs in the right-hand panel. *Significant difference compared with the untransfected control (*p* < 0.05). The values are presented as the mean ± S.E. for the number of myotubes shown in the parentheses of Table 4. The amount of Ca^2+^ in the SR was significantly increased by STIM2-knockdown.

To examine SERCA1a activity in the STIM2-knockdown myotubes, an oxalate-supported ^45^Ca^2+^-uptake assay was conducted. The Ca^2+^-uptake activity of SERCA1a was not changed at a resting cytosolic Ca^2+^ concentration (70 nM of free Ca^2+^, Fig. 3a) by the STIM2-knockdown. However, at a higher cytosolic Ca^2+^ concentration, such as that found during skeletal muscle contraction (1 μM of free ^45^Ca^2+^), the Ca^2+^-uptake activity of SERCA1a was significantly increased by the STIM2-knockdown (more than 50% increase compared with the untransfected or scrambled siRNA control, Fig. 3a, and Table 3). This suggests that STIM2 attenuates SERCA1a activity at micromolar cytosolic Ca^2+^ concentrations in skeletal muscle (i.e., during skeletal muscle contraction).

**Table 3:** ^45^Ca^2+^-uptake from the cytosol into the SR through SERCA1a in the STIM2-knockdown mouse primary skeletal myotubes.

^**45**^ **Ca** ^**2+**^ **-uptake** (nmole/mg/min)	Untransfected	Scrambled siRNA	STIM2-knockdown
At 70 nM free ^45^Ca^2+^	1.64 ± 0.15	1.70 ± 0.13	2.06 ± 0.11
At 1 μM free ^45^Ca^2+^	8.01 ± 1.30	8.10 ± 0.30	12.17 ± 0.61*

Either an untransfected or a scrambled siRNA-transfected control was used as a negative control. The results are presented as the mean ± S.E. of three independent experiments. *Significant difference compared with the untransfected control (*p* < 0.05).

To access the increased SERCA1a activity by the STIM2-knockdown from a different angle, the cytosolic Ca^2+^ level at rest and the amount of Ca^2+^ in the SR in the STIM2-knockdown myotubes were measured using single-myotube Ca^2+^-imaging experiments (Fig. 3b,c). First, the cytosolic Ca^2+^ level at rest was significantly reduced by the STIM2-knockdown (Fig. 3b and Table 4). This result agreed with the prediction that more cytosolic Ca^2+^ could be taken up into the SR by the increased activity of SERCA1a via the STIM2-knockdown, which, subsequently, lowers the cytosolic Ca^2+^ level. Second, to measure the amount of Ca^2+^ in the SR (that is, to estimate how much Ca^2+^ is releasable from the SR to the cytosol), the SR of the STIM2-knockdown myotubes was depleted with thapsigargin (TG) in the absence of extracellular Ca^2+^. Extracellular free Ca^2+^ allows the avoidance of extracellular Ca^2+^ entry and an assessment of the amount of Ca^2+^ exclusively in the SR (Fig. 3c and Table 4). There was a significant increase in the amount of Ca^2+^ in the SR via STIM2-knockdown. Thus, STIM2-mediated attenuation of SERCA1a activity could account for the balanced Ca^2+^ distribution between the cytosol and the SR at rest in skeletal muscle.

**Table 4:** Properties of the STIM2-knockdown mouse primary skeletal myotubes.

		Untransfected	Scrambled siRNA	STIM2-knockdown
Resting [Ca^2+^]_cytosol_, nM		72.75 ± 9.16 (108)	72.50 ± 5.02 (116)	56.17 ± 6.05* (113)
Releasable Ca^2+^ from the SR		1.00 ± 0.09 (83)	0.99 ± 0.08 (88)	1.30 ± 0.14* (81)
SOCE	Peak area	1.00 ± 0.11 (76)	1.01 ± 0.12 (79)	0.80 ± 0.08* (73)
	Slope (the rate of the increase)	1.00 ± 0.05 (20)	0.97 ± 0.04 (20)	0.82 ± 0. 04* (20)
KCl response		1.00 ± 0.08 (74)	1.03 ± 0.07 (88)	1.31 ± 0.08* (92)
Caffeine response		1.00 ± 0.09 (74)	1.02 ± 0.09 (88)	1.26 ± 0.11* (92)

The values, except for those of the cytosolic Ca^2+^ levels at rest, were normalized to the mean value of those from the untransfected controls. The values are presented as the mean ± S.E. for the number of myotubes shown in parentheses. *Significant difference compared with the untransfected control (*p* < 0.05).

### STIM2 in skeletal myotubes also contributes to SOCE

**Figure 4:**
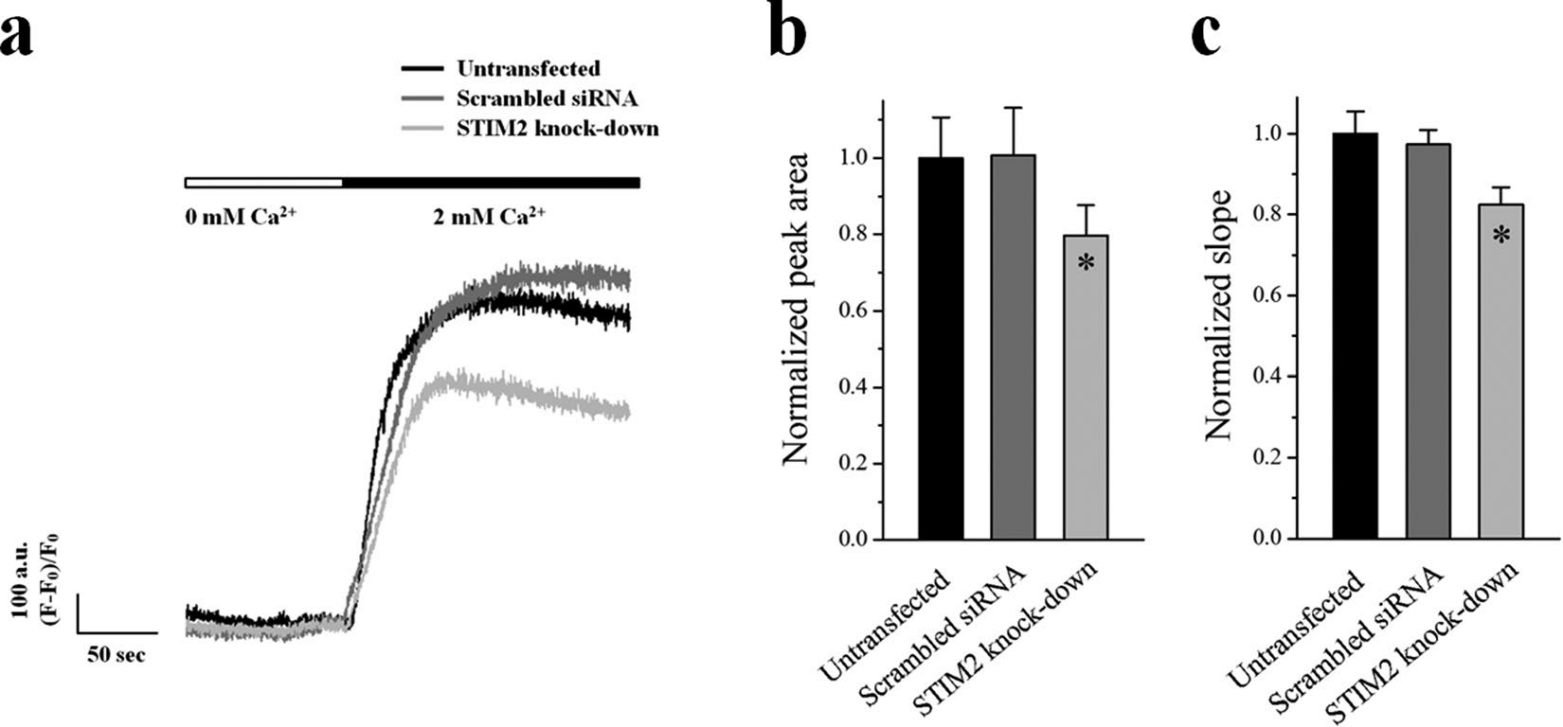
Decreased SOCE by STIM2-knockdown. The Ca^2+^ in the SR of the STIM2-knockdown myotubes was depleted by the treatment of TG (2.5 μ M) in the absence of extracellular Ca^2+^. Extracellular Ca^2+^ (2 mM) was applied to the myotubes to induce SOCE. (**a**) A representative trace for each group is shown. The results are summarized as bar graphs for the area under the peaks (**b**) or the slope at the rising phase of the peaks (**c**). *Significant difference compared with the untransfected control (*P* < 0.05). The values are presented as the mean ± S.E. for the number of myotubes shown in the parentheses of Table 4. SOCE was significantly decreased by STIM2-knockdown.

To examine the role of STIM2 in the SOCE of skeletal muscle, Ca^2+^ in the SR was depleted with TG in the absence of extracellular Ca^2+^ in the STIM2-knockdown myotubes, and extracellular Ca^2+^ was applied to the myotubes (Fig. 4a). Both the area under the peaks (i.e., the amount of Ca^2+^ entry, Fig. 4b) and the slope at the rising phase of the peaks (i.e., the rate of Ca^2+^ entry, Fig. 4c) that represent the overall degree of extracellular Ca^2+^ entry during SOCE were decreased by STIM2-knockdown. Therefore SOCE was significantly reduced via STIM2-knockdown (Fig. 4 and Table 4). This result suggests that, in addition to the primary role of STIM1 in the SOCE, STIM2 also contributes to SOCE in skeletal muscle.

It is possible that the STIM2-knockdown could alter the expression levels of Orai1 and STIM1 that mainly mediate SOCE in skeletal muscle, and their altered expression could be the cause of the reduced SOCE in the STIM2-knockdown myotubes. The expression levels of either Orai1 or STIM1 were accessed by immunoblot assay (see the results of the immunoblot assays below). There was no significant change in the protein content levels of either Orai1 or STIM1 via STIM2-knockdown. Therefore, the decrease in SOCE via STIM2-knockdown was not due to a simple change in the expression levels of either Orai1 or STIM1.

### STIM2 in skeletal myotubes regulates the intracellular Ca^2+^ release from the SR to the cytosol through RyR1 during skeletal muscle contraction

**Figure 5:**
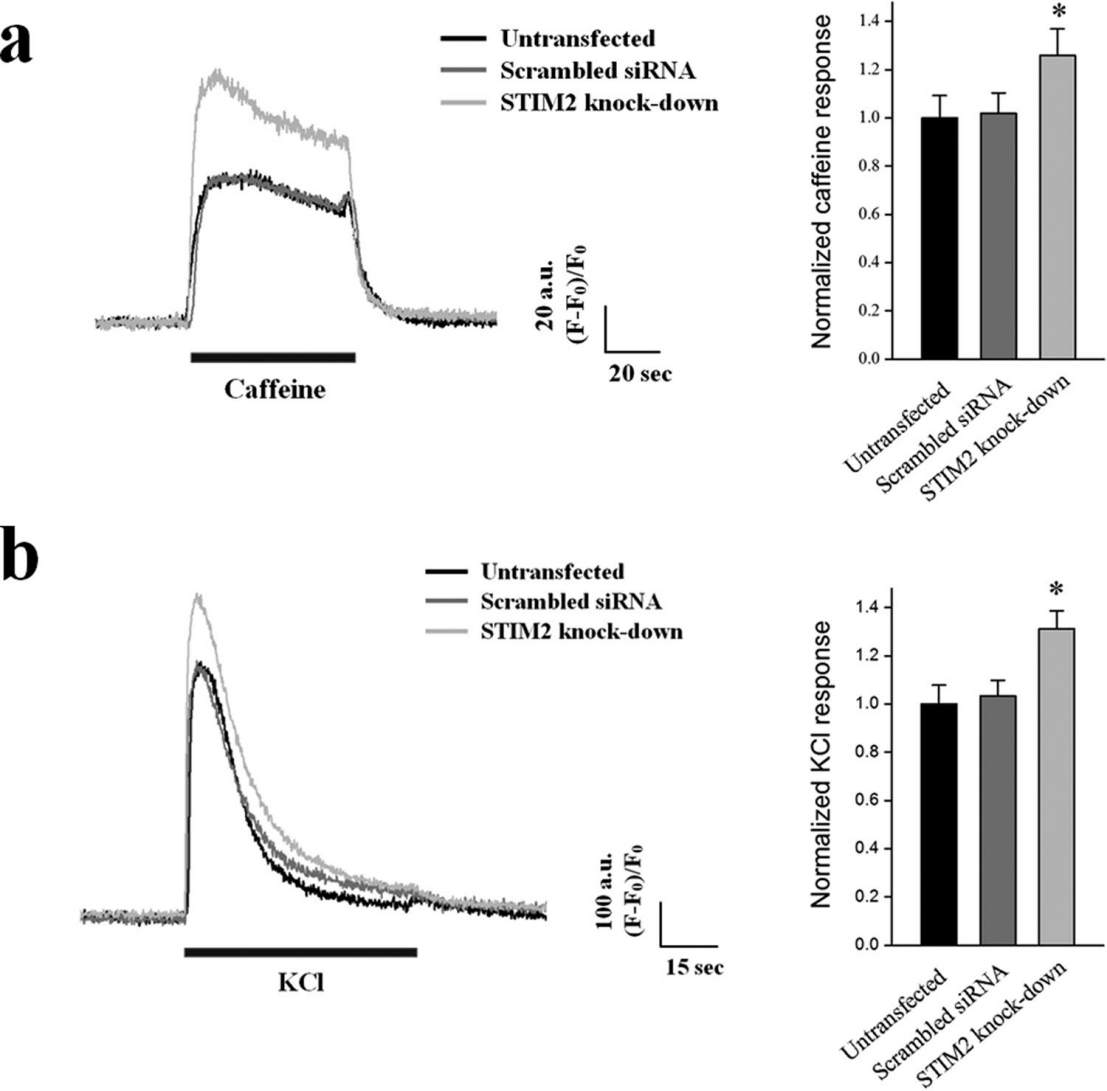
Enhanced intracellular Ca^2+^ release through RyR1 in response to caffeine or KCl by STIM2-knockdown. Caffeine (a specific agonist of RyR1) (**a**), or KCl (a membrane depolarizer) (**b**), was applied to the STIM2-knockdown myotubes, and the intracellular Ca^2+^ release from the SR to cytosol through RyR1 in the myotubes was measured. Histograms are shown for the normalized peak amplitude to the mean value of those from the untransfected control. The results are presented as the mean ± S.E. for the number of experiments in the parentheses in Table 4. *Significant difference was compared with the untransfected controls (*P* < 0.05). Intracellular Ca^2+^ releases in response to either caffeine or KCl is significantly increased by STIM2-knockdown.

To increase the cytosolic Ca^2+^ level to a certain point at a given time is the key step in skeletal muscle contraction, and RyR1 is the only channel that is responsible for intracellular Ca^2+^ release from the SR to the cytosol for skeletal muscle contraction. Intracellular Ca^2+^ releases through RyR1 in response to caffeine, a direct agonist of RyR1 in skeletal muscle^55^, were examined in the STIM2-knockdown myotubes using Ca^2+^-imaging experiments (Fig. 5a). Responses to caffeine were increased via STIM2-knockdown (Fig. 5a and Table 4). Responses to KCl, a membrane depolarizer (that can induce coupling between DHPR and RyR1 and then intracellular Ca^2+^ release from the SR to the cytosol through RyR1 for skeletal muscle contraction^1–4^, i.e., the mimicry of intracellular Ca^2+^ release during skeletal muscle contraction) were also increased by the STIM2-knockdown to an approximately equal response to caffeine (Fig. 5b, Table 4). Therefore, STIM2 in skeletal muscle participates in intracellular Ca^2+^ release during skeletal muscle contraction.

**Figure 6:**
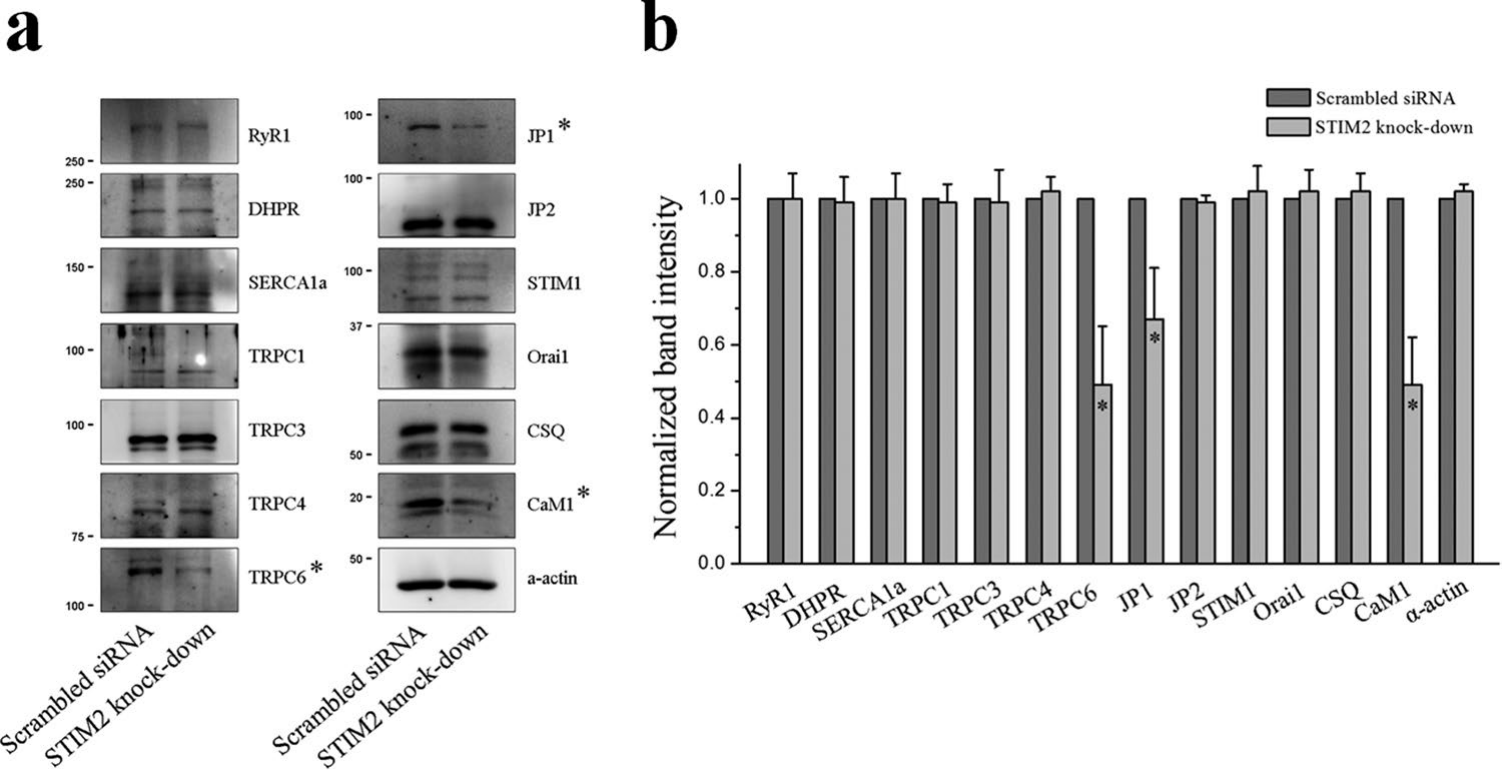
Decreased protein content level of TRPC6, JP1, or CaM1 by STIM2-knockdown. (**a**) Lysate of the STIM2-knockdown myotubes was subjected to an immunoblot assay with one of the antibodies against thirteen proteins that mediate or regulate skeletal muscle functions. *α*-Actin was used as a loading control. Three independent experiments per each protein were conducted. JP, junctophilin; CSQ, calsequestin. The immunoblot data were cropped from the immunoblot images of different gels and were grouped. The full-length blots are presented in Supplementary Figs 6 to 19. Among them, the expression level of TRPC6, JP1, or CaM1 was significantly decreased (indicated by asterisks). (**b**) The protein content level in (a) is presented as bar graphs. Bar graphs are presented as the mean ± S.E. for three independent experiments. *Significant difference compared with the scrambled siRNA control (*P* < 0.05). The protein content level of TRPC6, JP1, or CaM1 in the STIM2-knockdown myotubes was significantly decreased.

To find additional possible factors for the changes by the STIM2-knockdown above, thirteen proteins that mediate or regulate Ca^2+^ movements or Ca^2+^ handling in skeletal muscle were examined by immunoblot assays using the lysate of STIM2-knockdown myotubes (Fig. 6a). There was no significant change in the protein content levels of the three main proteins that mediate Ca^2+^ movements during the contraction and relaxation of skeletal muscle: DHPR, RyR1 and SERCA1a. No change in SERCA1a protein content suggests that the increased SERCA1a activity in the STIM2-knockdown myotubes was not simply due to an increase in the protein content levels of SERCA1a. There also was no significant change in the protein content levels of Orai1 and STIM1 or in the proteins that are responsible for extracellular Ca^2+^ entry in skeletal muscle, TRPC1, TRPC3, and TRPC4, but there was a significant decrease in the protein content levels of TRPC6 (Fig. 6b). There also was a significant decrease in the protein content levels of JP1, which mediates the proper formation of the junctional membrane complex (Fig. 6b). However, based on transmission electron microscopy observations, there was no significant change in the junctional membrane complex by the STIM2-knockdown (data not shown), suggesting that the reduced amount of JP1 was sufficient to properly form the junctional membrane complex. There was no change in the protein content levels of JP2 or calsequestrin. Interestingly, via the STIM2-knockdown, there was a significant decrease in the protein content levels of calmodulin 1 (CaM1), which is a ubiquitous Ca^2+^-binding protein in various cells (Fig. 6b). These results suggest that TRPC6 and CaM1 could be involved in the changes that were found in the STIM2-knockdown myotubes.

## Discussion

In the present study, we searched for STIM2-binding proteins from among proteins that mediate or regulate skeletal muscle functions, and we examined the functional role of the STIM2-binding protein along with STIM2 in skeletal muscle. STIM2 binds to SERCA1a via its C-terminal region of amino acids from 453 to 729, and attenuates SERCA1a activity during skeletal muscle contraction. It seems that STIM2 could be involved in a moderate level of Ca^2+^-uptake to the SR through SERCA1a by attenuating SERCA1a activity in order to maintain skeletal muscle contraction for longer periods.

During SOCE, three dimers of STIM1 (i.e., six STIM1s) are required to activate ‘a’ functional hexameric Orai1 that contains a central pore^56,57^. This stoichiometry seems not to be in the interaction between STIM2 and SERCA1a because a functional SERCA1a is monomeric and there is no symmetry in the three-dimensional structure of SERCA1a^58,59^. However, considering that STIM2 shares homologies with STIM1 in the amino acid sequences and domains, it is still possible that a dimeric or oligomeric STIM2 (possibly via EF-SAM, first coiled-coil domain, and/or CAD that are known to be involved in the self-oligomerization of STIM1^20,21,33^) participates in the interaction with a SERCA1a.

Previously, we reported that STIM1 binds to SERCA1a in skeletal muscle and is required for the full-activity of SERCA1a during skeletal muscle relaxation, which means STIM1 is a positive regulator of SERCA1a^45^. In the present study, we found another *in-situ* SERCA1a regulator in skeletal muscle, STIM2, but its regulation is the reverse of STIM1, which means STIM2 is a negative regulator of SERCA1a. The relationship between STIM1 and STIM2 in terms of regulating SERCA1a is akin to a tug-of war that results in SERCA1a that is neither too much nor too little.

Interruption of STIM1-SERCA1a interaction by an over-expression of the SERCA1a-binding region of STIM1 in mouse primary skeletal myotubes induced effects similar to the properties of STIM2-knockdown myotubes in the present study, and resulted in a decrease in cytosolic Ca^2+^ levels at rest, an increase in the amount of Ca^2+^ in the SR, but no effect on terminal differentiation^45^. However, under the disrupted interaction of STIM1 or STIM2 with SERCA1a, the regulation of intercellular Ca^2+^ movement through RyR1 in response to either caffeine or KCl differed: Ca^2+^ movement through RyR1 was decreased by a disrupted STIM1-SERCA1a interaction^45^, but it was increased by a disrupted STIM2-SERCA1a interaction, as shown via the use of STIM2-knockdown in the present study. The binding region of STIM1 or STIM2 to SERCA1a belongs to the variable region between STIM1 and STIM2 (amino acids from 449 to 671 in STIM1, and from 453 to 729 in STIM2), which possibly creates the differences in the roles of STIM1 and STIM2 in skeletal muscle. Different properties in the sensing of Ca^2+^ and in the self-oligomerization of STIM1 and STIM2 could also account for the different roles of STIM1 and STIM2 in skeletal muscle^39,40,60,61^. Considering that the regulation of SERCA2a activity by phospholamban depends on the phosphorylation status of phospholamban^62^, possible phosphorylation sites were searched in the SERCA1a-bindgin regions using NetPhos 3.1 (that predicts both generic- and kinase-specific phosphorylation sites), GPS 3.0 (that predicts kinase-specific phosphorylation sites in a proteome-wide level), or PhosphoSVM (that is a non-kinase-specific prediction tool) (Supplementary Fig. 2). There were no similarities between the predicted possible phosphorylation sites in the SERCA1a-binding regions of STIM1 and STIM2, in terms of the position, number, or pattern of the predicted phosphorylation sites. Therefore, it seems that the binding of STIM2 to SERCA1a could be mediated in a manner that differs from that of STIM1.

It has reported that the higher luminal Ca^2+^ content in the SR enhances the activity of RyR1 in mouse, frog, or rabbit skeletal muscle^63^. In addition, the presence of luminal Ca^2+^ in the SR is crucial for the coupled gating of RyR1 and DHPR during skeletal EC coupling^64,65^. Therefore the increase in the releasable Ca^2+^ content from the SR (which reflects the luminal Ca^2+^ content in the SR, Fig. 3c) is a clue to explain a possible mechanism whereby the intercellular Ca^2+^ movement through RyR1 in response to either caffeine or KCl is significantly increased by STIM2-knockdown. Meanwhile, unlike the results in the present study, STIM2-knockdown in human skeletal myotubes in a related study reduces the response to a membrane depolarizer such as KCl^66^. In that study, repeated KCl applications that mimicked the state of skeletal muscle fatigue were used, instead of a single KCl application that mimics a single twitch, which was used in the present study. Based on these two studies, it is possible that STIM2 could affect the response of RyR1 to membrane depolarization in a frequency-dependent manner.

Less is known about the roles of STIM2 in skeletal muscle. It is proposed that STIM1 and STIM2 are functionally redundant because the over-expression of either one of them corrects most of the defects on the terminal differentiation of human myoblasts to myotubes by STIM2- or STIM1-knockdown^43,66^. STIM2, however, is required for the terminal differentiation of human myoblasts to myotubes^66^. On the other hand, no significant change in the terminal differentiation by either STIM1- or STIM2-knockdown was found in either the present study (Fig. 2b) or in our previous research^45^. These discrepancies in the effects of STIM2 on terminal differentiation seem to be caused by different experimental procedures that were used to knock down STIM2. In the present study, instead of the siRNA transfection to myoblasts (i.e., totally undifferentiated forms) that was used by others, siRNA for STIM2 was transfected to immature myotubes on differentiation day 3 (Fig. 2b), because we tried to examine the interplay between STIM2 and SERCA1a, and SERCA1a was expressed from the middle to the end stage during terminal differentiation^67^. Taken together, these studies provide interesting input and show that, in skeletal muscle, STIM2 could be a multiplayer in a stage-dependent manner, both as a member for the terminal differentiation at the early stage of the terminal differentiation, and as a regulator of SERCA1a and a SOCE-mediator at the late, or mature, stage of terminal differentiation.

Ca^2+^-charged CaM1 (Ca^2+^-CaM1) exerts an inhibitory effect on RyR1 channel activity^68,69^. Less availability of Ca^2+^ due to decreases in SOCE by the STIM2-knockdown in the present study could induce a switch of Ca^2+^-CaM1 to aop-CaM1 that could enhance RyR1 activity. In addition, it is possible that the decreased protein content level of CaM1 by the STIM2-knockdown (Fig. 6a) could have reduced the availability of Ca^2+^-CaM1, which could also enhance RyR1 activity, and this could be a way to compensate for the reduced cytosolic Ca^2+^ level caused by the STIM2-knockdown. This is well supported by a previous report showing that CaM1 regulates cytosolic Ca^2+^ levels by regulating Ca^2+^ release from the SR in skeletal muscle^69^. Indeed, RyR1 activity in response to a specific agonist of RyR1, caffeine, was increased in the present study (Fig. 5a). CaM1 is known to bind to STIM2^70^. Therefore, our results again support the finding that STIM2 regulates cytosolic Ca^2+^ levels in skeletal muscle, and suggest the possibility that the regulation of cytosolic Ca^2+^ levels by STIM2 occurs in conjunction with CaM1.

In addition to the major role of Orai1 as a Ca^2+^ entry channel during SOCE^4,71^, canonical-type transient receptor potential cation channels (TRPC) have also been proposed as Ca^2+^ entry channels for SOCE in skeletal muscle^72–74^. Skeletal muscle expresses mainly four types of TRPCs: TRPC1, TRPC3, TRPC4, and TRPC6^75^. TRPC1, TRPC3, and TRPC4 are known to mediate SOCE in skeletal muscle^76,77^. However, little is known about TRPC6 in skeletal muscle, although it is reported that myoblasts from *mdx* mice, a mouse model of Duchenne muscular dystrophy, shows a reduced expression of TRPC6^78^. In the present study, TPRC6 protein content was significantly decreased by the STIM2-knockdown (Fig. 6), and STIM2 bound to TRPC6 in skeletal muscle (Supplementary Fig. 3). This suggests the possibility that TRPC6 could also have participated in the reduction of SOCE or in reduced cytosolic Ca^2+^ levels at rest via the STIM2-knockdown. However, the role of TRPC6 along with STIM2 in skeletal muscle should be resolved with a clear working mechanism.

Here we suggest that similar and different roles of STIM1 and STIM2 exist in the skeletal muscle. First, both STIM1 and STIM2 are responsible for SOCE, albeit possibly for different reasons. Second, STIM2 is only now being thought of as an *in-situ* regulator of SERCA1a. The regulatory activity of STIM2 on SERCA1a is the reverse of STIM1^26^. Third, the differences in the roles of STIM1 and STIM2 in intracellular Ca^2+^ movements in skeletal muscle could attribute to divergent and coordinated Ca^2+^-handling in skeletal muscle. Therefore, STIM2 could be a necessary partner of STIM1 in skeletal muscle function, and skeletal muscle could utilize STIM1 and/or STIM2, depending on the cellular context or need.

## Materials and Methods

### Ethics statement

All surgical interventions including pre- and post-surgical animal care were carried out in accordance with the Laboratory Animals Welfare Act, the Guide for Care and Use of Laboratory Animals, and the Guidelines and Policies for Rodent Survival Surgery approved by the Institutional Animal Care and Use Committee of the College of Medicine at The Catholic University of Korea. All experimental protocols were approved by the Committee of the College of Medicine at The Catholic University of Korea.

### cDNA construction, and protein expression of GST-fused STIM2-UI region

Human STIM2 cDNA was obtained from Addgene (Cambridge, MA, USA, #18862). To prepare the cDNA for the STIM2-UI region, oligonucleotide primers were designed on the basis of human STIM2 (GenBank accession number: NM_ AF328905.1) (Table 2, bottom panel). With these primers, PCR was performed (30 cycles at 95 °C for 45 s, 63 °C for 45 s, and 68 °C for 90 s). The PCR fragments were sub-cloned into pGEX-4T-1 vector with EcoR 1 and Sal I enzyme sites. The sequence of the construct was confirmed (ABI Prism 3700 DNA Sequencer, Applied Biosystems, ThermoFisher Scientific, Waltham, MA, USA). GST-fused STIM2-UI protein was expressed in E. coli (DH5α) using 0.1 mM isopropyl-β-D-thiogalactopyranoside (Sigma-Aldrich, St. Louis, MO, USA), as previously described^79,80^.

### Preparation of a triad sample, and binding assay of GST-fused STIM-UI protein with the triad sample

The triad vesicles that are enriched portions with proteins mediating or regulating skeletal muscle functions, including SERCA1a^3,4,81^, were prepared and solubilized to obtain the triad sample, as previously described^45,81,82^. Binding assays were performed, as previously described^45,80,83,84^. Briefly, affinity beads were prepared by immobilizing GST-fused STIM2-UI proteins on GST beads (Amersham, GE Healthcare Biosciences, Pittsburgh, PA). The affinity beads were then incubated with 150 μg of the solubilized triad sample for 8 h at 4 °C. The proteins that were bound to the affinity beads were separated on a 10% SDS-PAGE gel, and the gel was stained with Coomassie Brilliant Blue in order to obtain the protein bands.

### In-gel digestion, protein identification by qTOF-MS, and database search

Protein bands obtained from the binding assay were subjected to in-gel digestion with trypsin^80,81^. The digested peptide solution was desalted and concentrated, and was eluted using a homemade C18 nano-column (100–300 nl with trypsin of POROS reverse-phase R2 material (20–30 μm in bead size, PerSeptive Biosystems, Foster City, CA)) and 1.5 ul of 50% MeOH, 49% H_2_O, and 1% HCO_2_H. qTOF-MS of the eluted peptides was performed using a Hybrid Quadrupole-TOF LC/MS/MS Mass Spectrometer (AB Sciex Instruments, Framingham, MA) equipped with an electrospray ionization (ESI) source. The quadrupole analyzer was used to select precursor ions for fragmentation in the hexapole collision cell. The produced ions were analyzed using an orthogonal TOF analyzer and fitted with a reflector, a micro-channel plate detector, and a time-to-digital converter.

### Cell culture

Mouse primary skeletal myoblasts were derived from mouse skeletal muscle using a single-cell cloning method, and these proliferated and differentiated to myotubes, as previously described^15,26,45,83,85–87^. For the terminal differentiation of the primary skeletal myoblasts to myotubes, myoblasts were re-plated either on 10-cm plates (either for the preparation of myotube lysate or for an oxalate-supported ^45^Ca^2+^-uptake experiment) or on 96-well plates (for the single-myotube Ca^2+^ imaging experiments) coated with Matrigel (BD Biosciences, San Jose, CA, USA). All reagents for the cell cultures were obtained from Invitrogen (ThermoFisher Scientific).

### Knockdown of STIM2 in mouse primary skeletal myotubes

Three different siRNAs for mouse STIM2 (GenBank accession number NM_001081103.2) were selected using two forms of siRNA design software from Ambion (ThermoFisher Scientific) and from Sigma-Aldrich (Table 2, upper panel). A scrambled siRNA was used as a negative control. Immature myotubes on differentiation day 3 were transfected with one of the siRNAs in a mixture containing 600 μl of low-glucose DMEM, 60 *μ*l of X-tremeGENE siRNA Transfection Reagent (Roche Applied Science, Penzberg, Upper Bavaria, Germany), and 200 nM of the specific synthetic siRNAs, as previously described^15,26,45^. After the transfection, fully differentiated myotubes on differentiation day 5 were subjected to further experiments.

### Co-immunoprecipitation and immunoblot assays

For the co-immunoprecipitation assay, the solubilized triad sample (50 µg of total protein) was incubated with anti-STIM2 antibody (Sigma-Aldrich) overnight at 4 °C, as previously described^26,80,81,83^. Anti-STIM1 (Abcam, Cambridge, MA, USA), anti-SERCA1a (Thermo Scientific Inc., Rockford, IL, USA), or anti-TRPC6 (Alomone Laboratories, Jerusalem 9104201, Israel) antibody was used for immunoblot assay. For the immunoblot assay, fully differentiated mouse primary skeletal myotubes on 10-cm plates on differentiation day 5 were solubilized, and the solubilized lysate (5 or 10 μg of total protein) was subjected to SDS-PAGE (8, 10, or 12% gel) and immunoblot assay, as previously described^15,26,45,80,83,87^. Anti-RyR1, anti-CSQ, anti-CaM1, anti-JP1, and anti-JP2 antibodies were obtained from Thermo Scientific Inc. Anti-TRPC1, anti-TRPC3, and anti-TRPC4 antibodies were obtained from Alomone Laboratories. Anti-DHPR, anti-Orai1, and anti-α-actin antibodies were obtained from Abcam. Horseradish peroxidase-conjugated anti-goat, anti-mouse, or anti-rabbit secondary antibodies were obtained from Jackson ImmunoResearch Laboratories (West Grove, PA, USA). The membranes were washed three times with PBS and developed using a SuperSignal Ultra Chemiluminescent substrate (Pierce, Rockford, IL, USA).

### Oxalate-supported ^45^Ca^2+^-uptake experiment

The STIM2-knockdown myotubes were homogenated in a buffer (50 mM KH_2_PO_4_, 10 mM NaF, 1 mM EDTA, 0.3 M sucrose, protease inhibitor cocktail (Roche Applied Science), and 0.5 mM DTT at pH 7.0) with a homogenizer for 15 s at speed 5 (IKA T10basic Ultra-turrax, Wilmington, NC, USA)^45,80^. Then, 250 μg of the myotube homogenate was subjected to an oxalate-supported ^45^Ca^2+^-uptake experiment. Briefly, the reaction buffer was composed of 40 mM imidazole, 100 mM KCl, 5 mM MgCl_2_, 5 mM NaN_3_, and 0.5 mM EGTA at pH 7.0. The washing buffer was composed of 100 mM KCl and 20 mM MOPS at pH 7.0. The uptake reaction was begun by the rapid sequential addition of 5 mM Mg-ATP, 5 mM K-oxalate, and either 70 nM or 1 μM of free ^45^Ca^2+^ (Perkin- Elmer, Waltham, MA, USA). The rate of ^45^Ca^2+^ uptake was calculated from the linear regression of ^45^Ca^2+^ uptake at 0, 1, 2, 3, and 4 min.

### Single-myotube Ca^2+^ imaging experiment

Single-myotube Ca^2+^ imaging experiments were performed using an inverted stage microscope (Nikon Eclipse TS100, Melville, NY, USA) equipped with a 40X oil-immersion objective (NA 1.30), a high-speed monochromator with a 75 W xenon lamp (FSM150Xe, Bentham Instruments, Verona, VA, USA), and a 12-bit charge-coupled device camera (DVC-340M-OO-CL, Digital Video Camera Company, Austin, TX 78744, USA), as previously described^15,26,45,81,83,88^. Fully differentiated mouse primary skeletal myotubes on 96-well plates were loaded with 5 μM fura-2-AM (for the measurement of the cytosolic Ca^2+^ levels at rest, Invitrogen, ThermoFisher Scientific) or with 5 μM fluo-4-AM (for other measurements, Invitrogen, ThermoFisher Scientific) in an imaging buffer (25 mM Hepes, pH 7.4, 125 mM NaCl, 5 mM KCl, 2 mM KH_2_PO_4_, 2 mM CaCl_2_, 6 mM glucose, 1.2 mM MgSO_4_, and 0.05% BSA) at 37 °C for 45 min. The data were displayed and analyzed using image acquisition and analysis software (High-Speed InCyt Im1 or Im2, v5.29, Intracellular Imaging Inc., Cincinnati, OH, USA). Either KCl or caffeine was dissolved in the imaging buffer and was applied via an auto-perfusion system (AutoMate Scientific, Berkeley, CA, USA). To measure the amount of Ca^2+^ in the SR, TG (2.5 μM, dissolved in DMSO, < 0.05%) was applied to the myotubes in the absence of extracellular Ca^2+^ in order to avoid extracellular Ca^2+^ entry. DMSO (0.05%) alone had no effect on the release of Ca^2+^. For the measurement of SOCE, the SR Ca^2+^ was depleted with TG (2.5 μM) in the absence of extracellular Ca^2+^, and once the cytosolic Ca^2+^ level returned to the baseline, 2 mM Ca^2+^ was added to the myotubes to measure the SOCE. To analyze the Ca^2+^ release obtained from the Ca^2+^ imaging experiments, the peak amplitude, which exhibited similar increases or decreases in peak areas, was considered. For long-term Ca^2+^ releases such as SOCE and TG responses, the areas under the curves were analyzed. For the analysis of SOCE, the slope at the rising phase of the peaks (i.e., the rate of SOCE) was also examined by a linear equation that was obtained from a linear fitting of the rising phase of the peaks.

### In silico approach

The possible phosphorylation site was predicted using NetPhos 3.1^89^, GPS 3.0^90^, or PhosphoSVM^91^. Under NetPhos 3.1, both generic- and kinase-specific predictions are performed, and the kinase-specific predictions by NetPhos 3.1 cover the prediction by NetPhosK 1.0. GPS 3.0 predicts kinase-specific phosphorylations with a large-scale prediction of >13,000 mammalian phosphorylation sites and a proteome-wide prediction of Aurora-B specific substrates including protein-protein interaction information. PhosphoSVM is a non-kinase-specific prediction tool which detects possible phosphorylation sites for which the associated kinase is unknown or the number of known substrate sequences of the associated kinase is few. For the prediction using NetPhos 3.1 or PhosphoSVM, predictions with more than 0.7 in score were considered. For the predictions using GPS 3.0, threshold was set at high and predictions with more than 20 in score were considered.

### Statistical analysis

The results are presented as the mean ± S.E. for the number of myotubes shown in the parentheses of Tables or in the legends of either the Tables or Figures. The values were normalized to the mean value from the corresponding controls. The significant differences were analyzed using an unpaired *t*-test (GraphPad InStat, v2.04, GraphPad Software, La Jolla, CA, USA). The differences were considered to be significant at *p < *0.05. The graphs were prepared using Origin v7 software.

## Electronic supplementary material

Supplementary Figures
