# Supplementary material for: STIM2 regulates both intracellular Ca2+ distribution and Ca2+ movement in skeletal myotubes
Source: Sci Rep. 2017 Dec 20;7:17936. doi: 10.1038/s41598-017-18256-3 (PMC5738411; doi:10.1038/s41598-017-18256-3)

STIM2 regulates both intracellular Ca^{2+} distribution and Ca^{2+} movement in skeletal myotubes

Mi Ri Oh, Keon Jin Lee, Mei Huang, Jin Ock Kim, Do Han Kim, Chung-Hyun Cho, and Eun Hui Lee

(SREP-17-38455)

Supplementary Figure 1. MS spectrums for bands 1 to 7 in Figure 1c and Table 1

(a) MS spectrum of the band #1 in Figure 1c and Table 1.

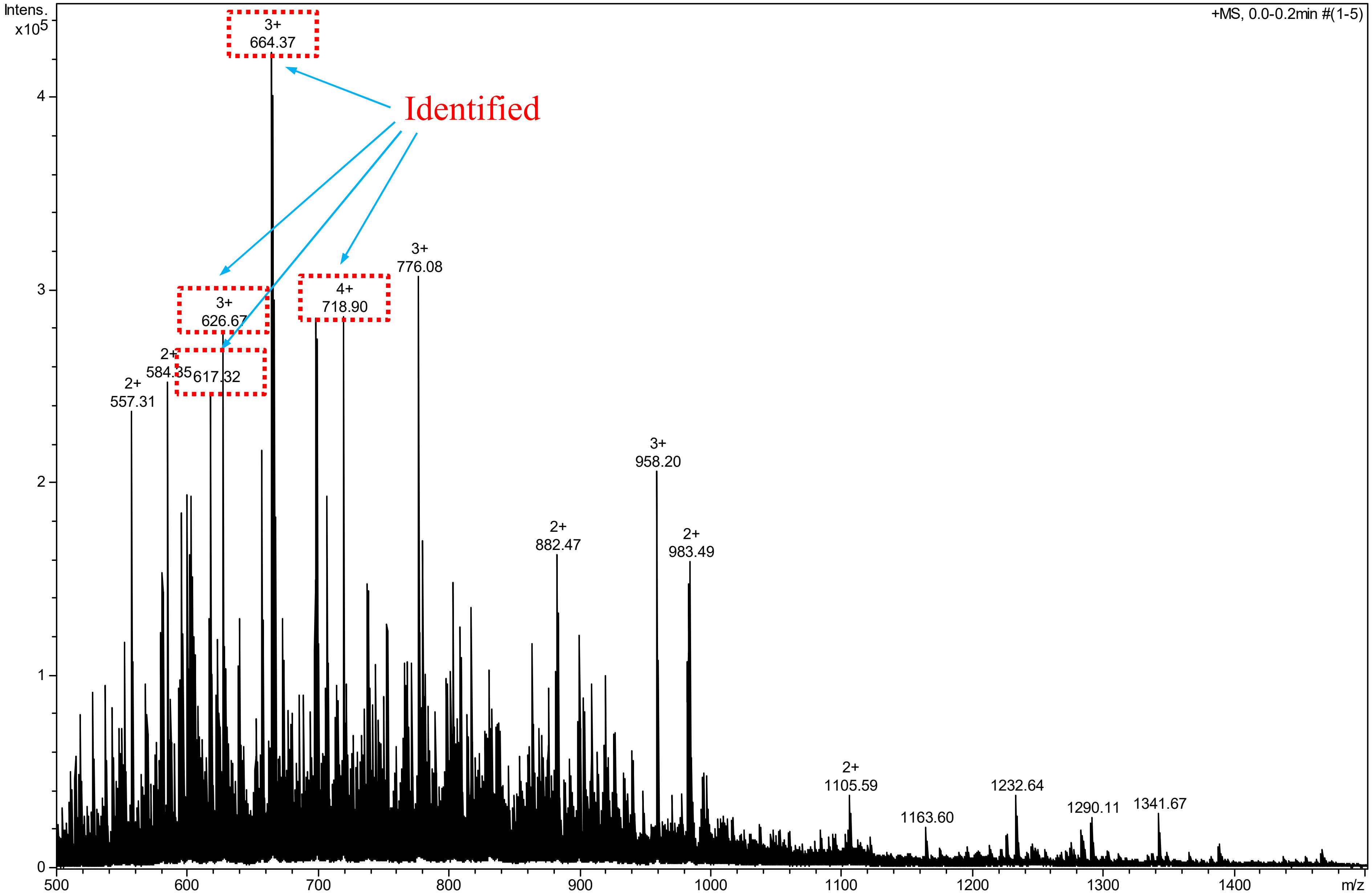

(b) MS spectrum of the band #2 in Figure 1c and Table 1.

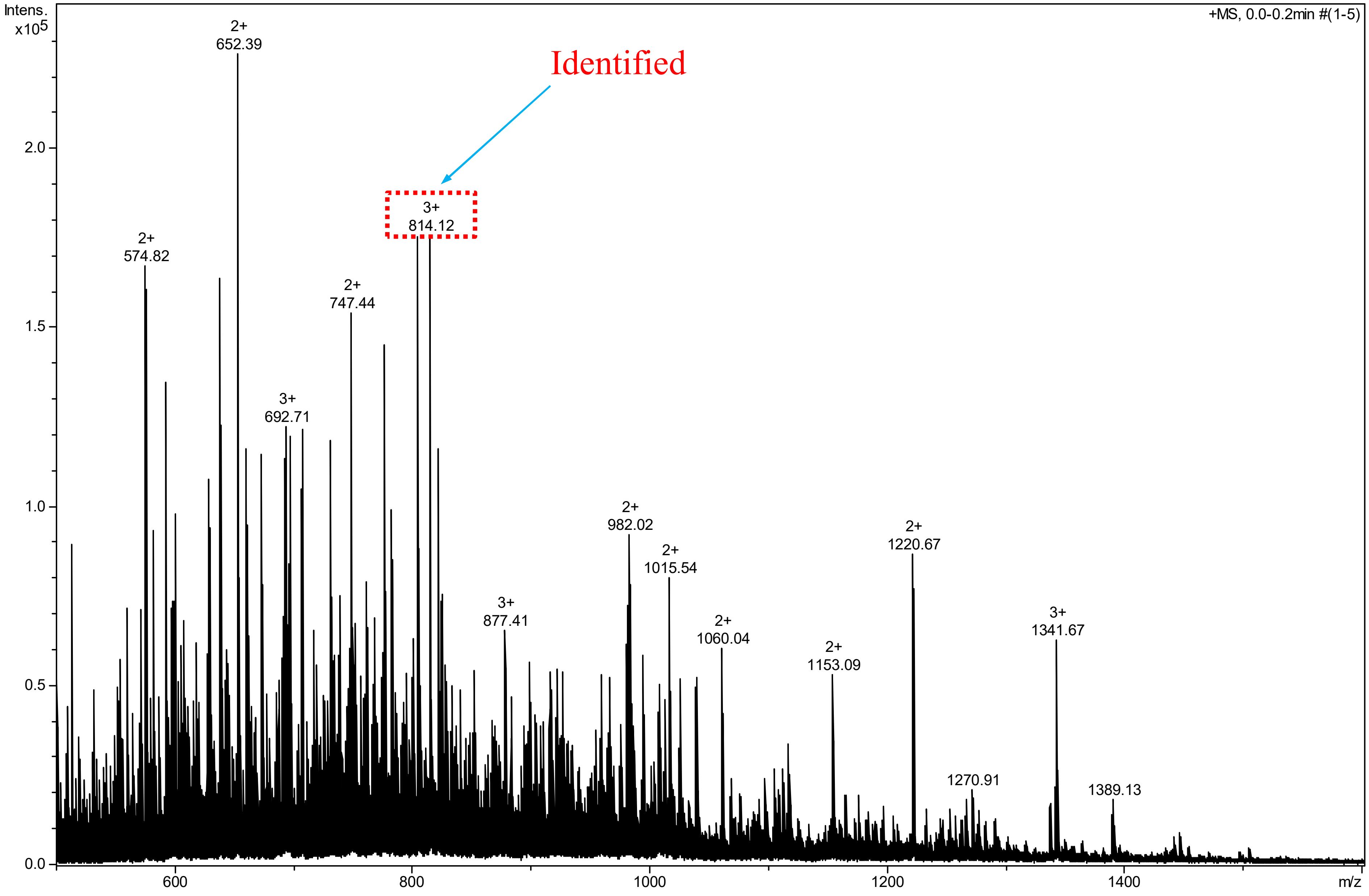

(c) MS spectrum of the band #3 in Figure 1c and Table 1.

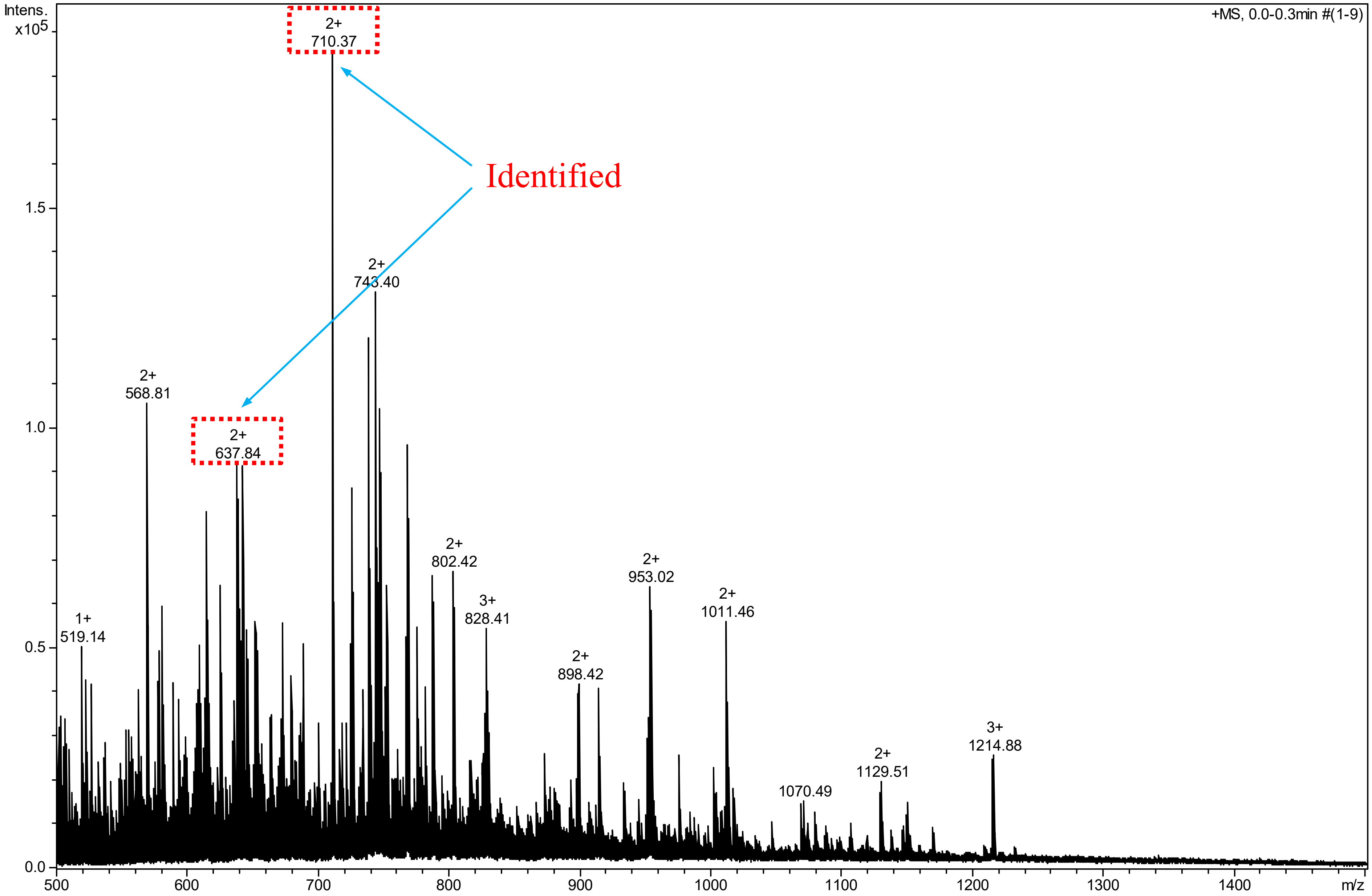

(d) MS spectrum of the band #4 in Figure 1c and Table 1.

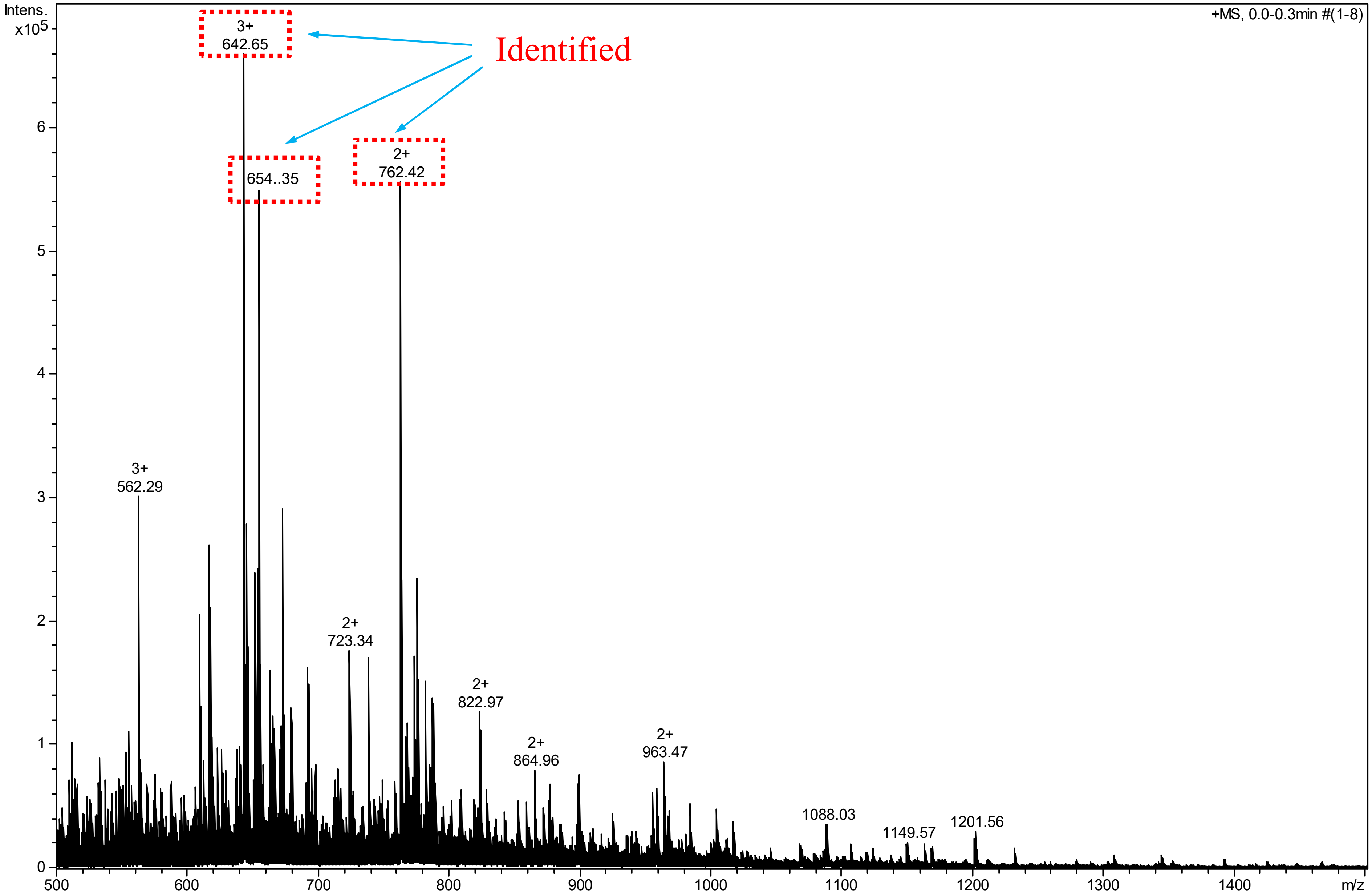

(e) MS spectrum of the band #5 in Figure 1c and Table 1.

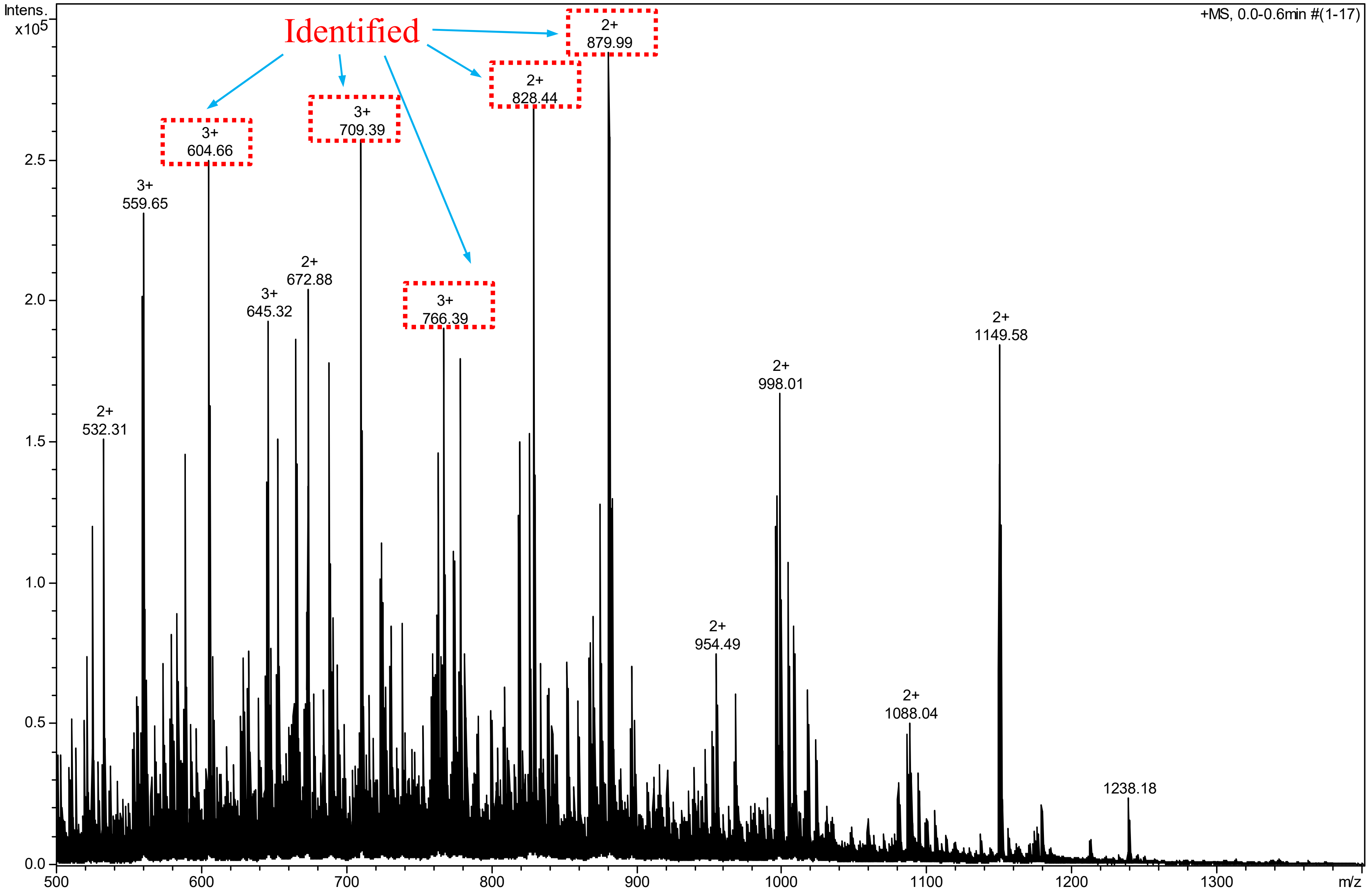

(f) MS spectrum of the band #6 in Figure 1c and Table 1.

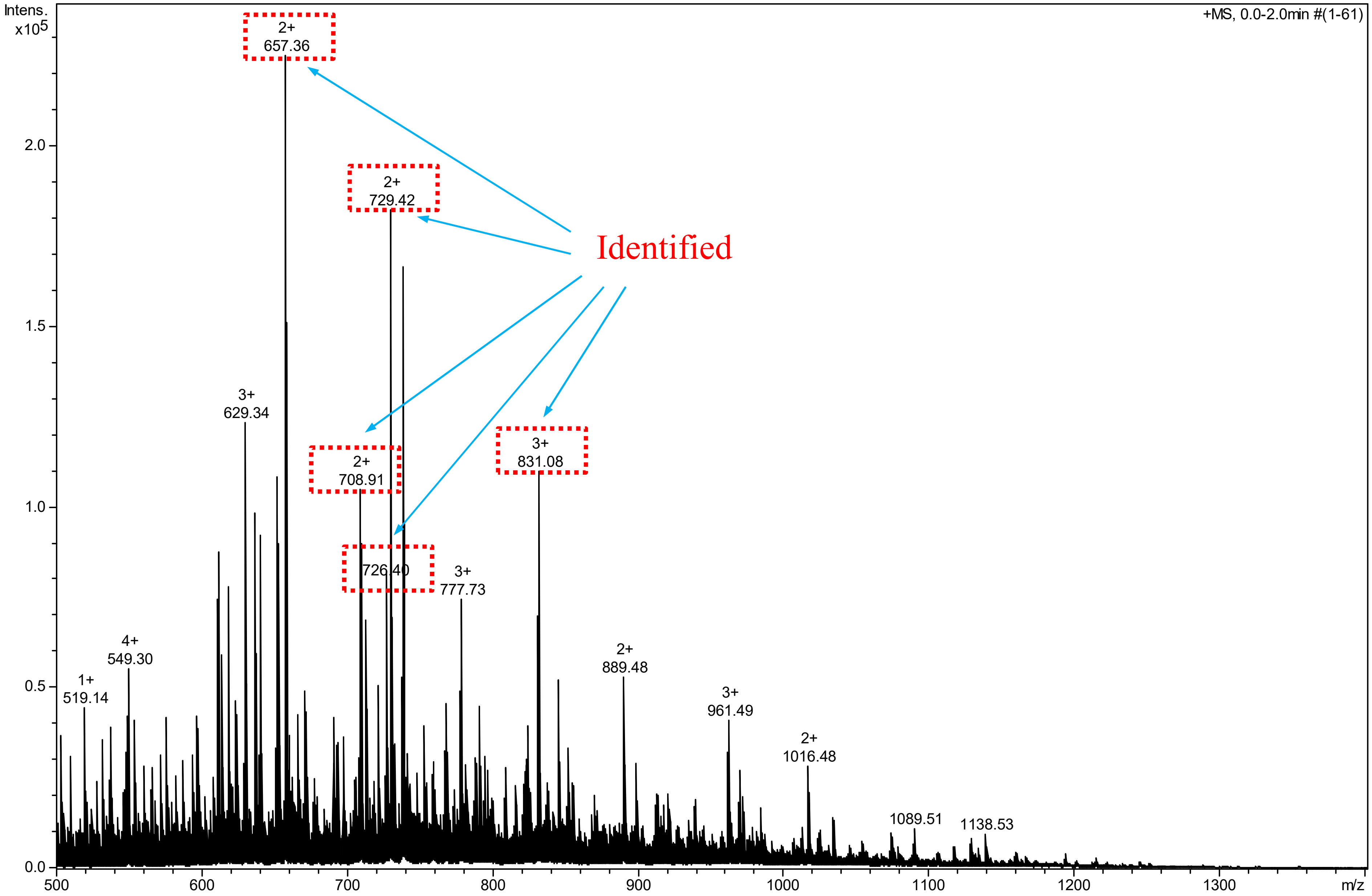

(g) MS spectrum of the band #7 in Figure 1c and Table 1.

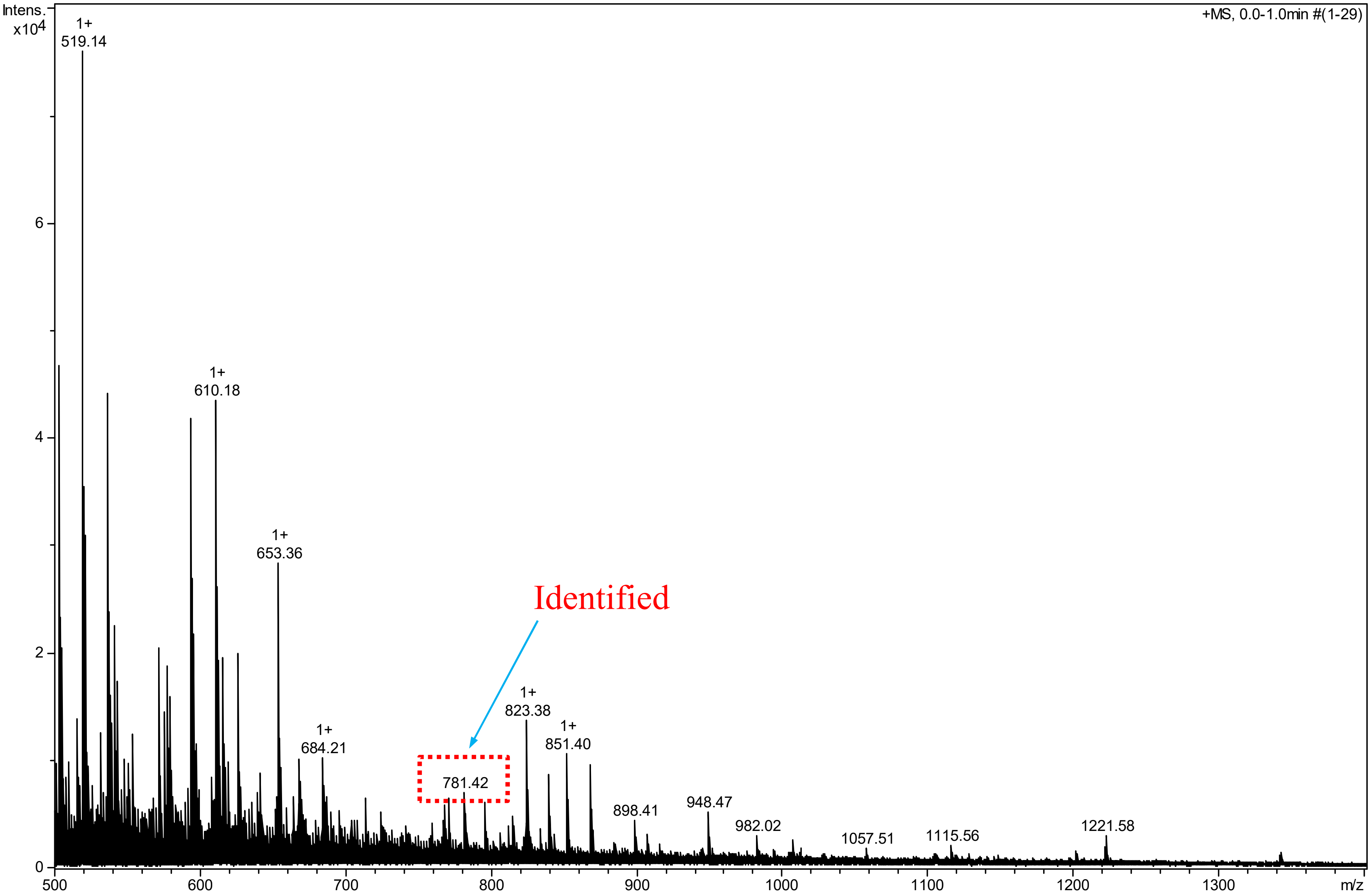

Supplementary Figure 2

The possible phosphorylation sites in the SERCA1a-binding regions of STIM1 and STIM2

The possible phosphorylation sites in the SERCA1a-binding regions of STIM1 **(a)** and STIM2 **(b)** were predicted using NetPhos 3.1, GPS 3.0, or PhosphoSVM. Under NetPhos 3.1, both generic- and kinase-specific predictions are performed, and the kinase-specific predictions by NetPhos 3.1 cover the prediction by NetPhosK 1.0. GPS 3.0 predicts kinase-specific phosphorylation with a large-scale prediction of >13,000 mammalian phosphorylation sites and a proteome-wide prediction of Aurora-B specific substrates including protein-protein interaction information. PhosphoSVM is a non-kinase-specific prediction tool which detects possible phosphorylation sites for which the associated kinase is unknown or the number of known substrate sequences of the associated kinase is few. For the prediction using NetPhos 3.1 (■) or PhosphoSVM (▲), the predictions with more than 0.7 in score were considered. For the prediction using GPS 3.0 (●), the threshold was set at high and the predictions with more than 20 in score were considered. Three digit numbers in the beginning or end of the amino acid sequences indicate the position of the SERCA1a-binding region in either STIM1 or STIM2. The predicted phosphorylation sites by all three software were colored in red. **(c)** The number of possible phosphorylation sites in the SERCA1a-binding regions in (a) and (b) were summarized. S, T, or Y means serine, threonine, or tyrosine, respectively. There were no similarities between the predicted possible phosphorylation sites in the SERCA1a-binding regions, in terms of the position, number, or pattern of the predicted phosphorylation sites.

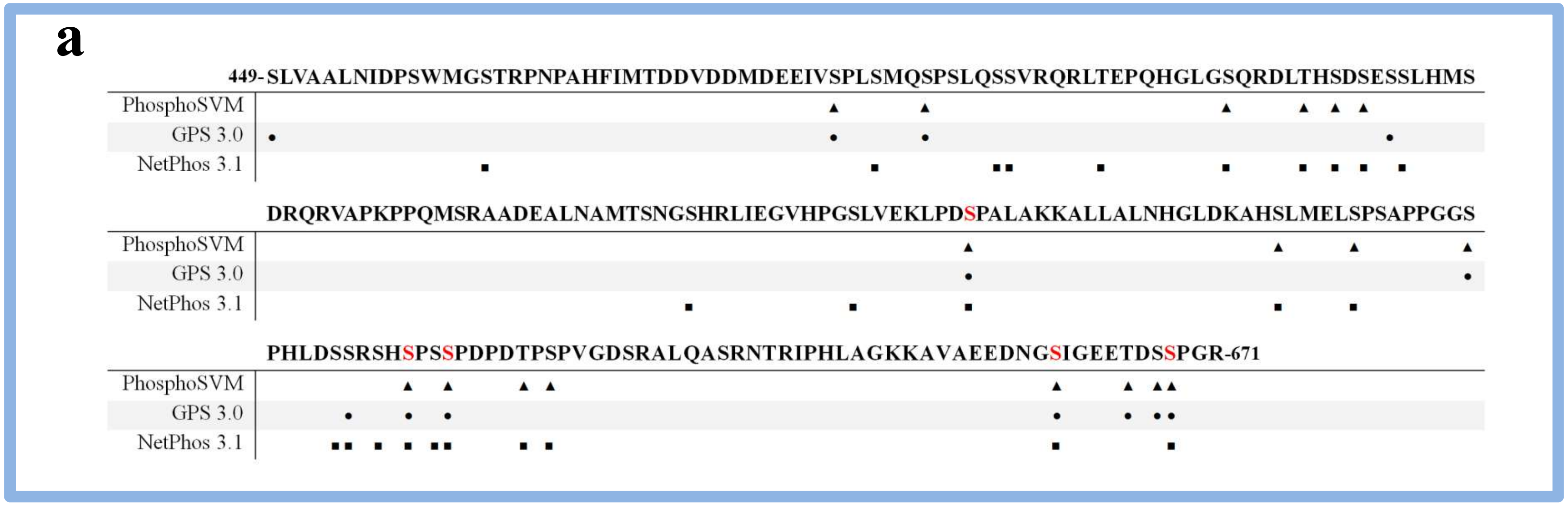

Supplementary Figure 2 (continued)

b

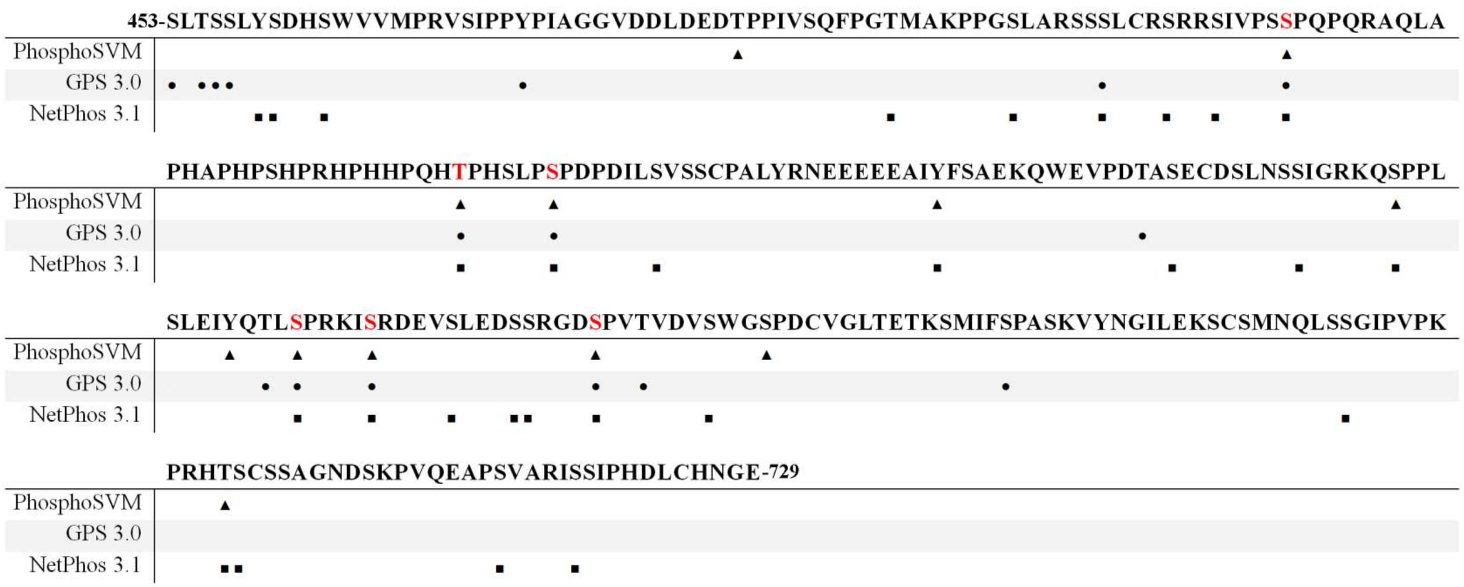

c

Phosphorable amino acid	PhosphoSVM		GPS 3.0		NetPhos 3.1	
	STIM1	STIM2	STIM1	STIM2	STIM1	STIM2
S	15	7	12	10	22	23
T	3	3	1	5	3	3
Y	0	2	0	1	0	2

Supplementary Figure 3

Co-immunoprecipitation of STIM2 with TRPC6

The triad sample obtained from rabbit skeletal muscle (30 μg of total proteins) was subjected to a co-immunoprecipitation assay with anti-STIM2 antibody, and the immunoprecipitant was subjected to immunoblot analysis with anti-STIM2 or anti-TRPC6 antibodies. Triad sample indicates the simple immunoblot of the triad sample. Without Ab indicates a reaction without anti-STIM2 antibody. IB, IP, or Ab means immunoblot, immunoprecipitation, or antibody, respectively. TRPC6 was co-immunoprecipitated with STIM2.

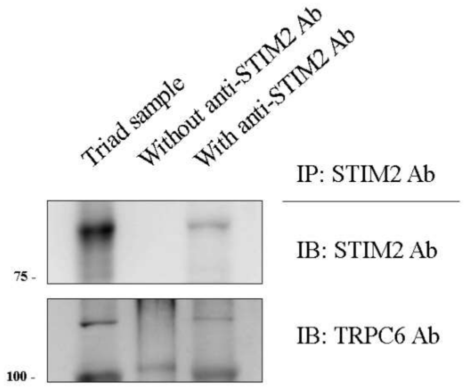

Supplementary Figure 4. The full-length blots for STIM2 and SERCA1a in Figure 1d.

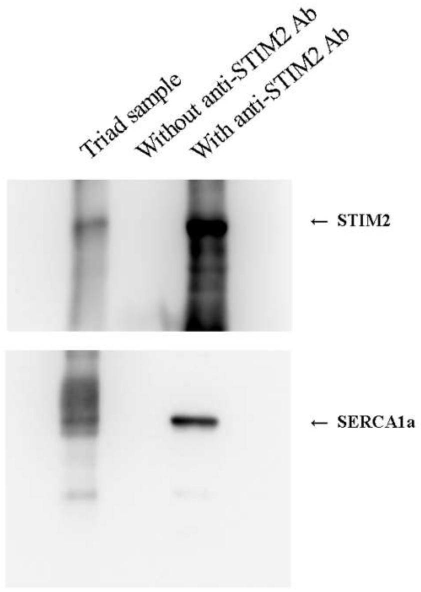

Supplementary Figure 5. The full-length blots for STIM2 and α-actin in Figure 2a.

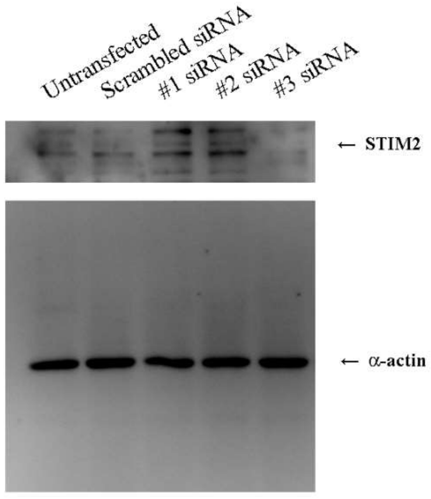

Supplementary Figure 6. The full-length blot for RyR1 in Figure 6a.

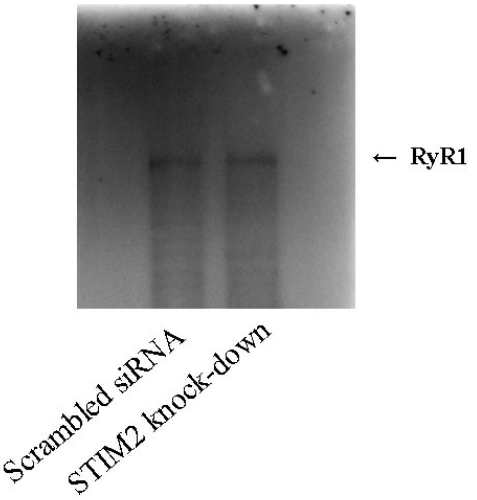

Supplementary Figure 7. The full-length blot for DHPR in Figure 6a.

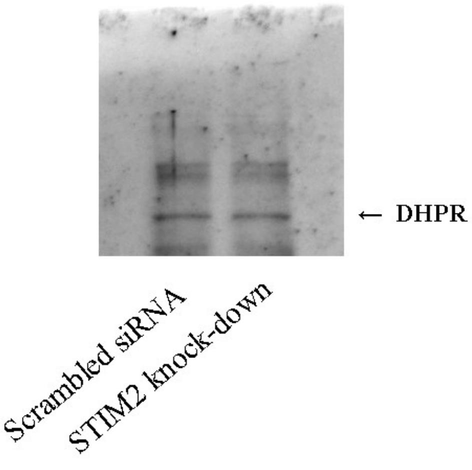

Supplementary Figure 8. The full-length blot for SERCA1a in Figure 6a.

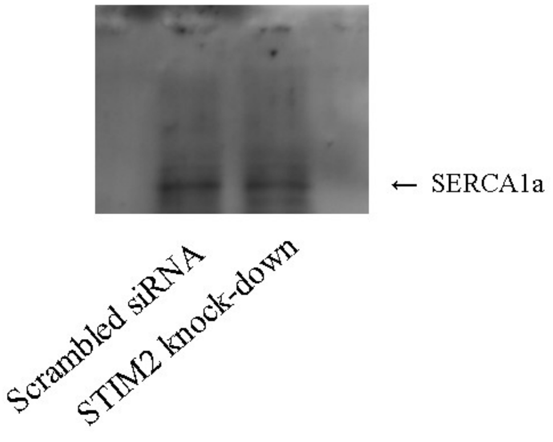

Supplementary Figure 9. The full-length blot for TRPC1 in Figure 6a.

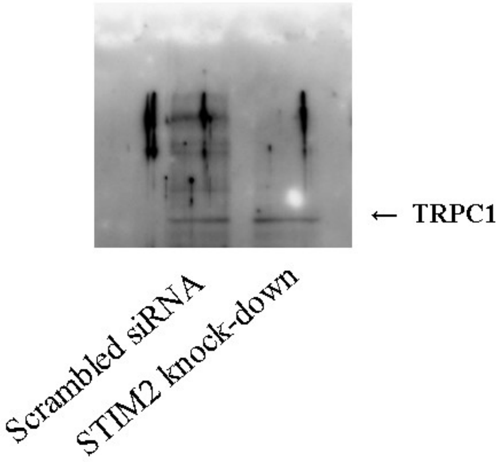

Supplementary Figure 10. The full-length blot for TRPC3 in Figure 6a.

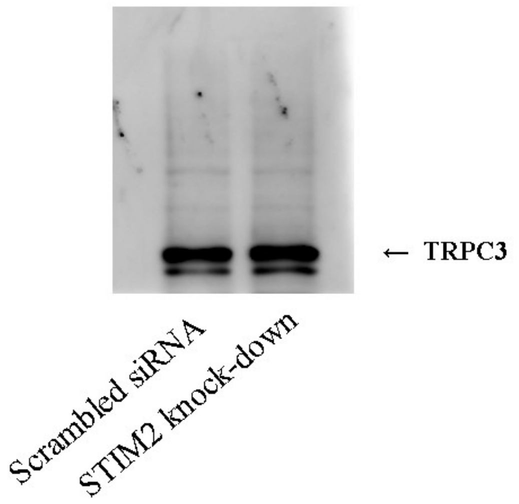

Supplementary Figure 11. The full-length blot for TRPC4 in Figure 6a.

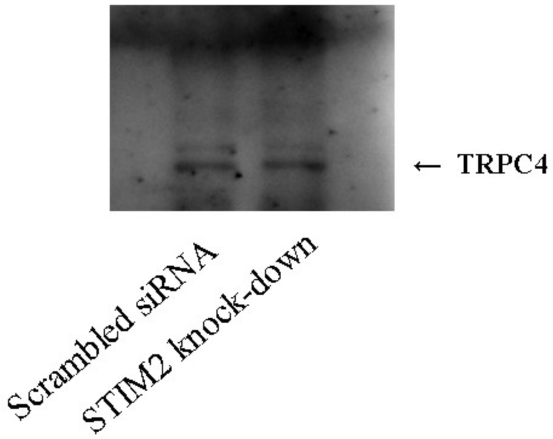

Supplementary Figure 12. The full-length blot for TRPC6 in Figure 6a.

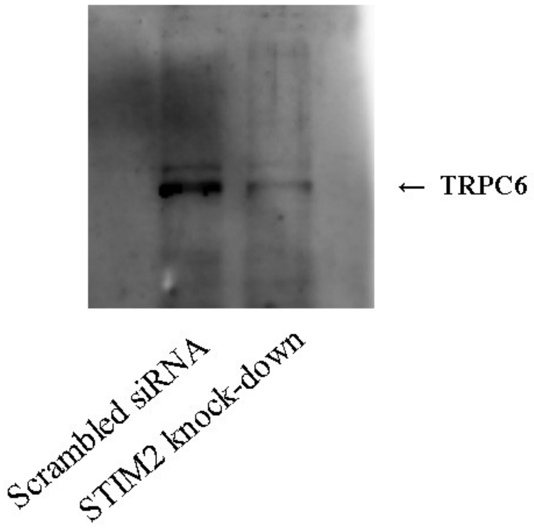

Supplementary Figure 13. The full-length blot for JP1 in Figure 6a.

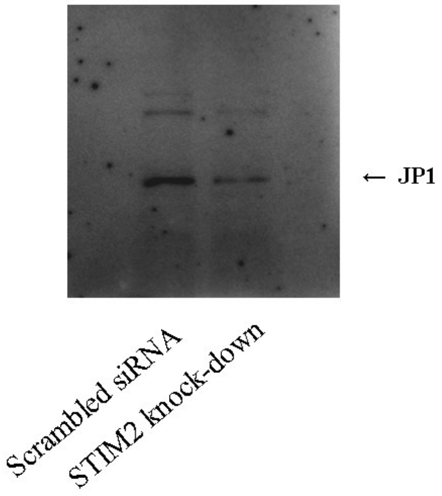

Supplementary Figure 14. The full-length blot for JP2 in Figure 6a.

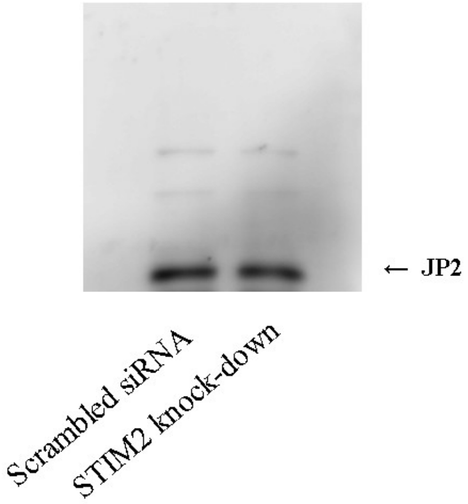

Supplementary Figure 15. The full-length blot for STIM1 in Figure 6a.

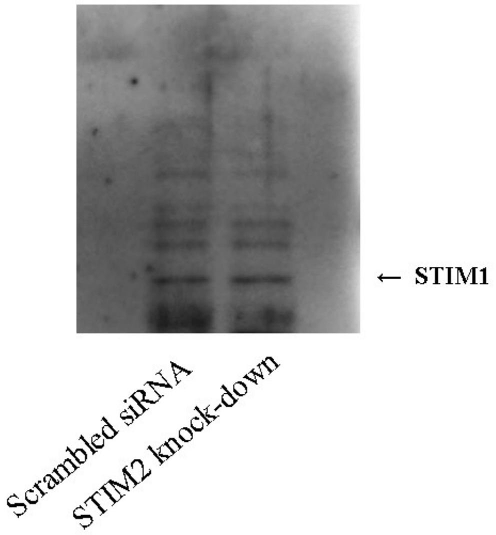

Supplementary Figure 16. The full-length blot for Orai1 in Figure 6a.

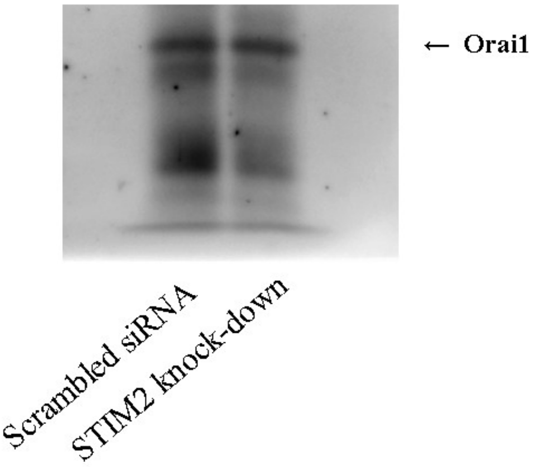

Supplementary Figure 17. The full-length blot for CSQ in Figure 6a.

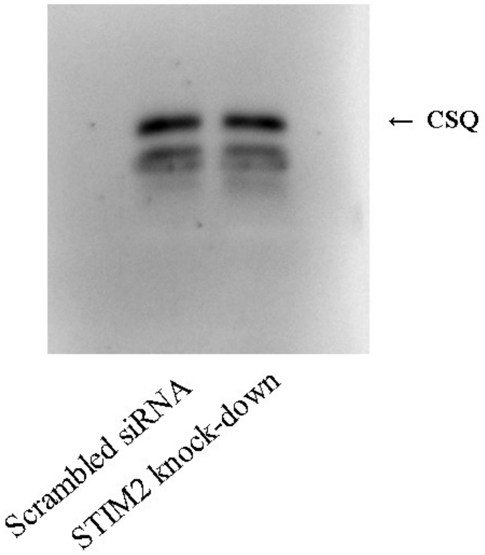

Supplementary Figure 18. The full-length blot for CaM1 in Figure 6a.

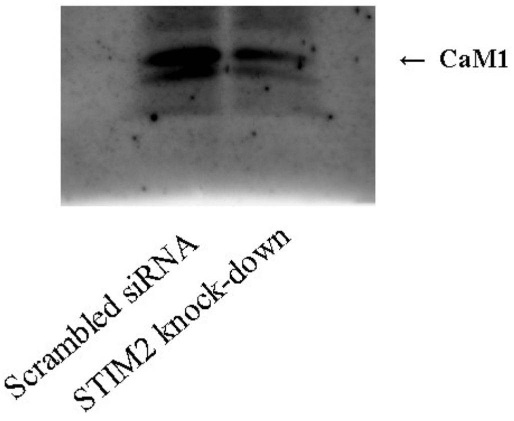

Supplementary Figure 19. The full-length blot for α-actin in Figure 6a.

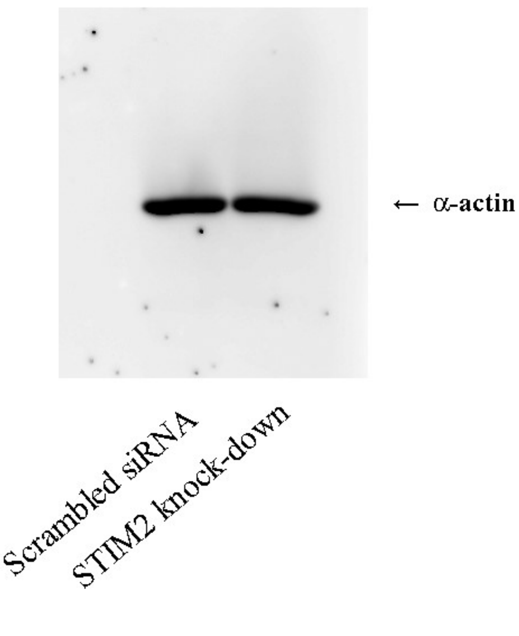

Supplement: Supplementary file 1 — Supplementary Figures [file 41598_2017_18256_MOESM1_ESM.pdf]
